# Supplementary figures and images for: Loss of Mir146b with aging contributes to inflammation and mitochondrial dysfunction in thioglycollate-elicited peritoneal macrophages
Source: eLife. 2021 Aug 23;10:e66703. doi: 10.7554/eLife.66703 (PMC8412946; doi:10.7554/eLife.66703)

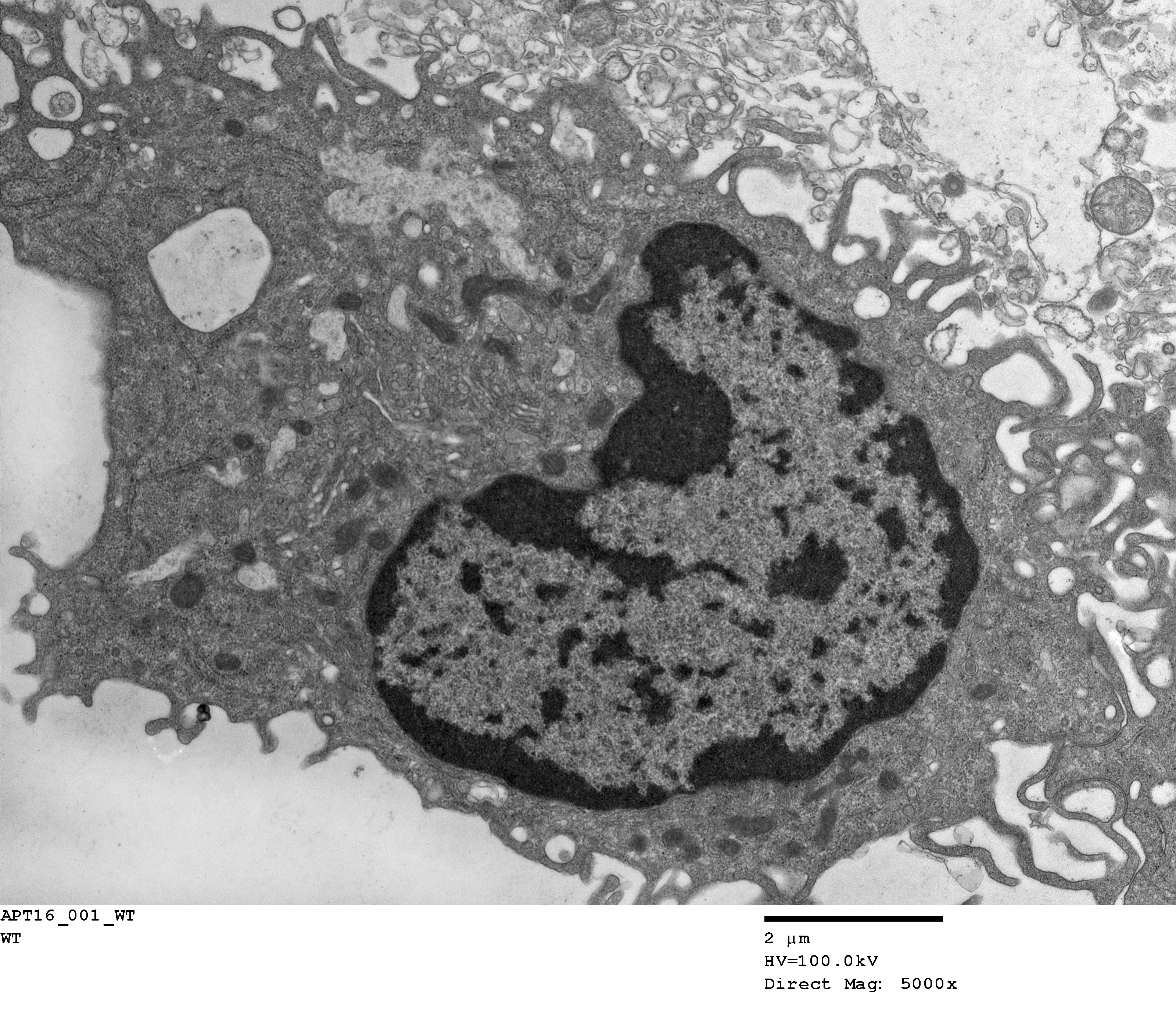

Supplement: Figure 3—source data 1. [file elife-66703-fig3-data1.zip › control EM Pt1 Fig3ABDE/APT16_001_WT.TIF]

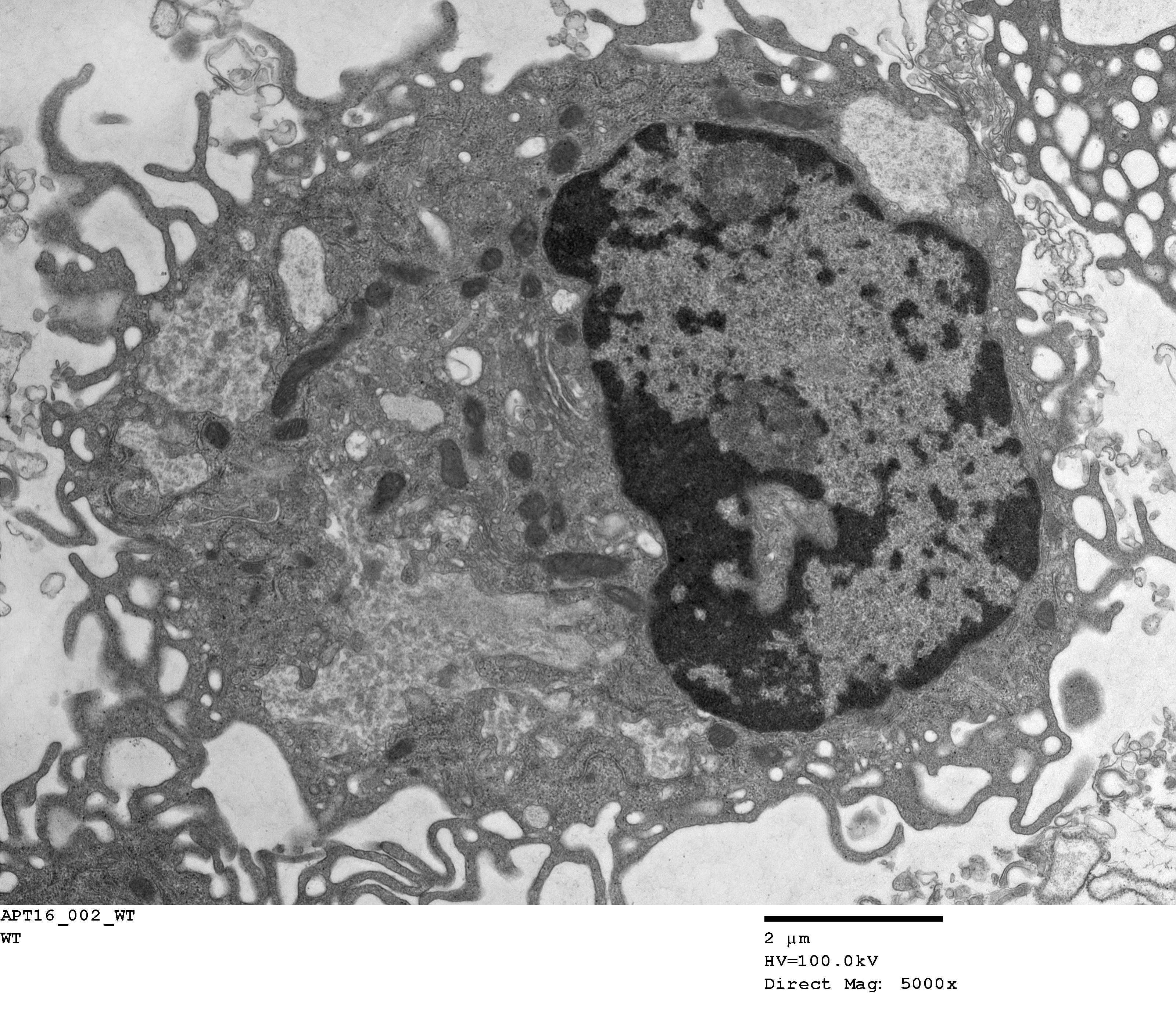

Supplement: Figure 3—source data 1. [file elife-66703-fig3-data1.zip › control EM Pt1 Fig3ABDE/APT16_002_WT.TIF]

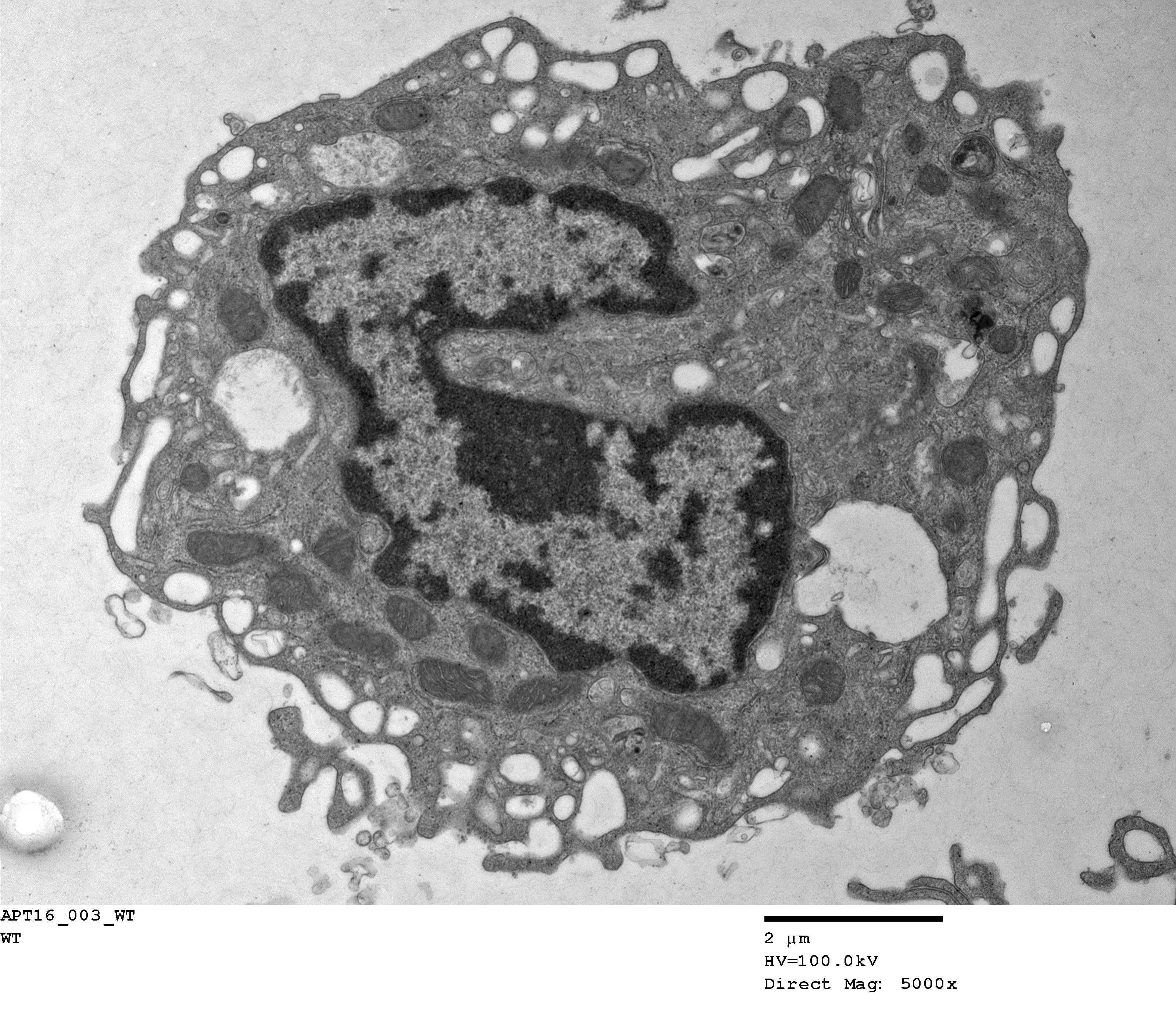

Supplement: Figure 3—source data 1. [file elife-66703-fig3-data1.zip › control EM Pt1 Fig3ABDE/APT16_003_WT.TIF]

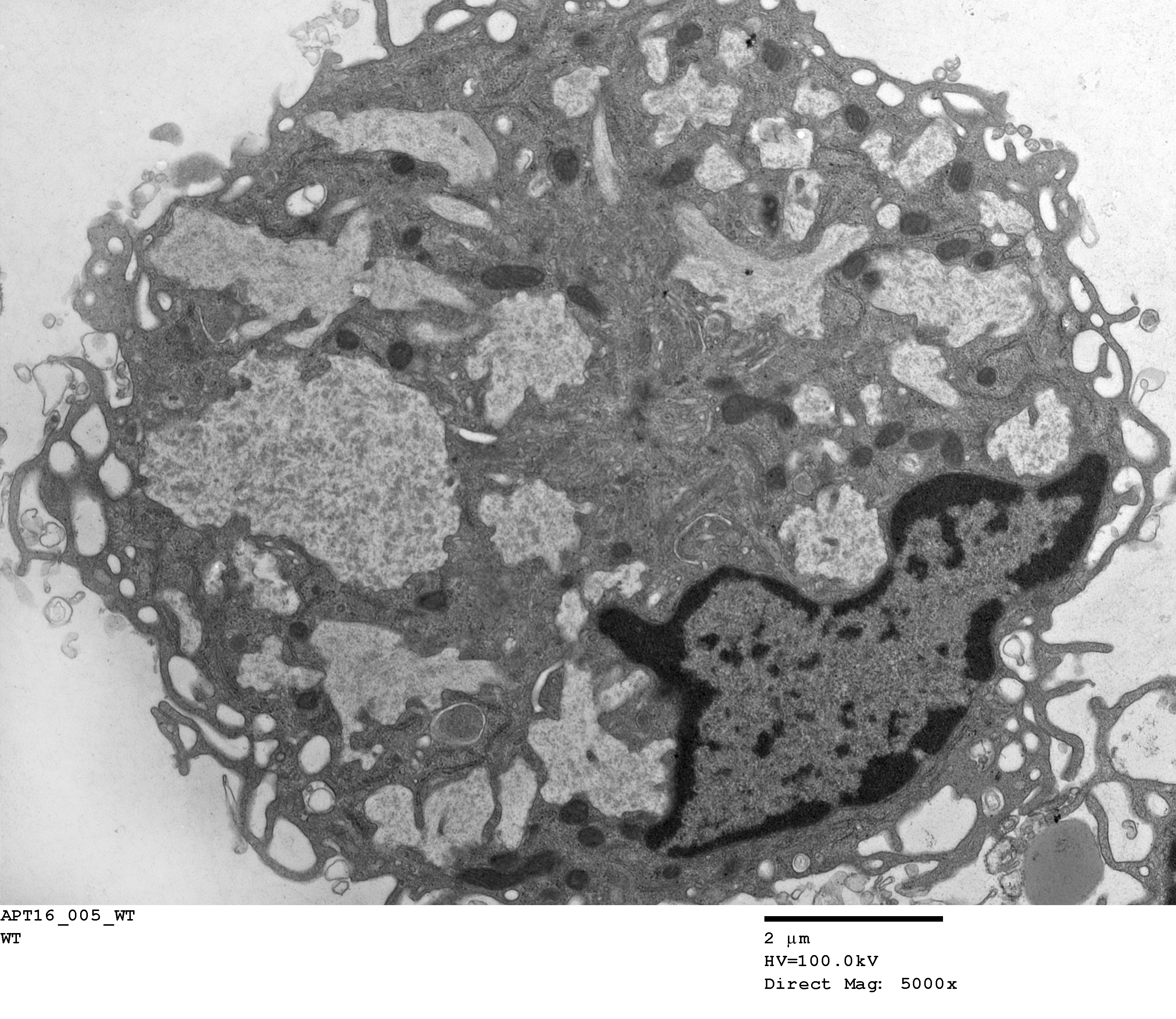

Supplement: Figure 3—source data 1. [file elife-66703-fig3-data1.zip › control EM Pt1 Fig3ABDE/APT16_005_WT.TIF]

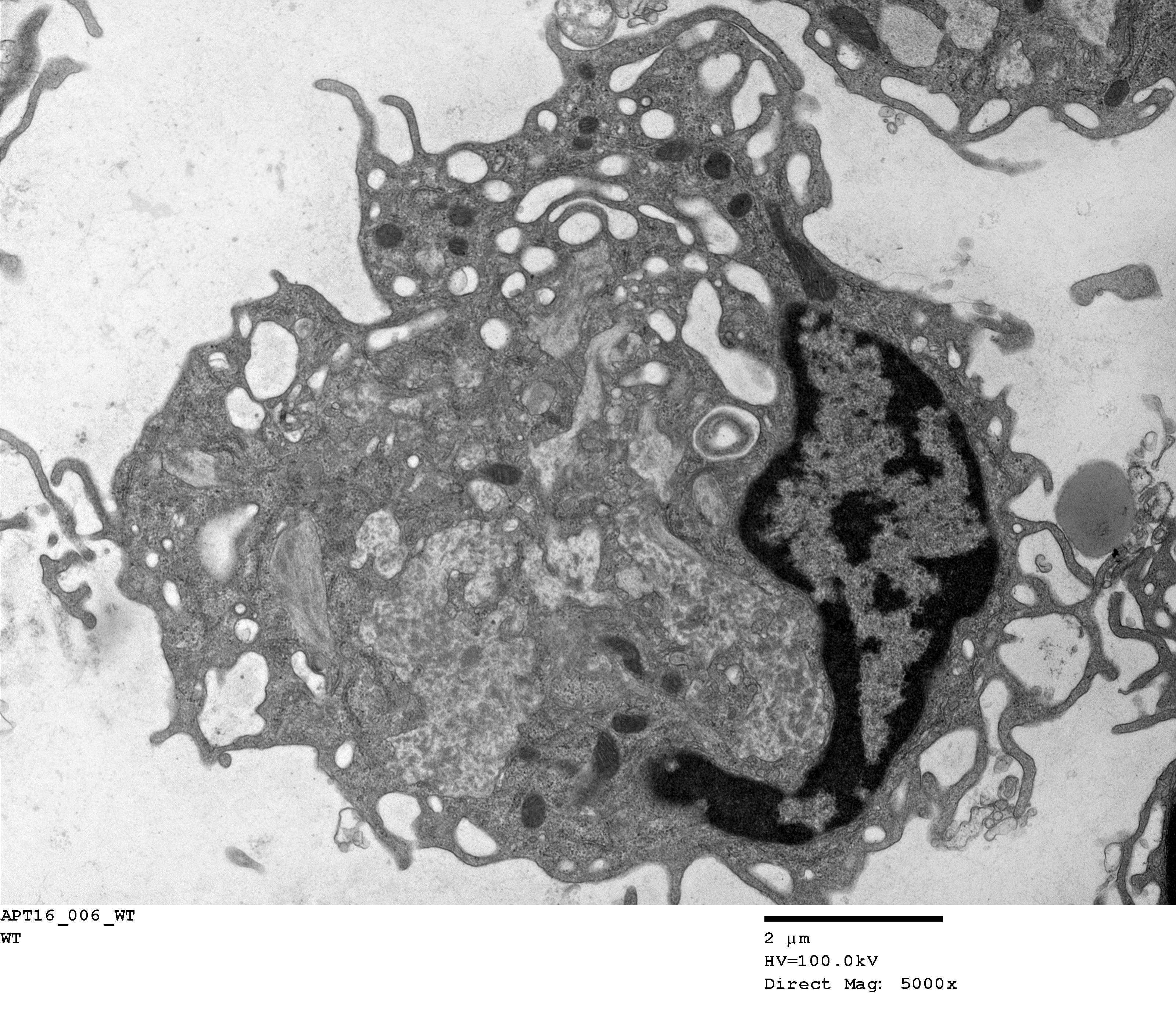

Supplement: Figure 3—source data 1. [file elife-66703-fig3-data1.zip › control EM Pt1 Fig3ABDE/APT16_006_WT.TIF]

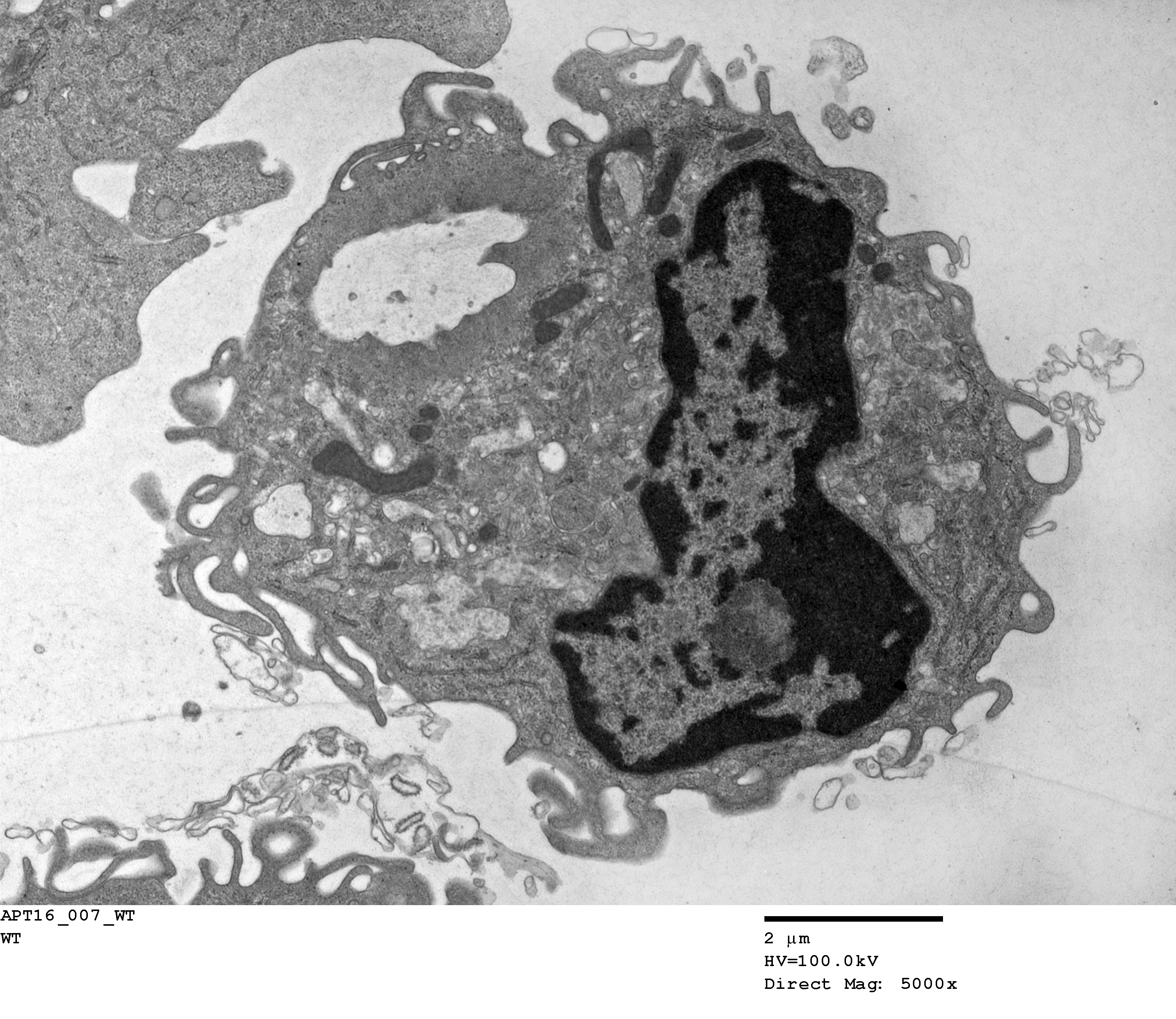

Supplement: Figure 3—source data 1. [file elife-66703-fig3-data1.zip › control EM Pt1 Fig3ABDE/APT16_007_WT.TIF]

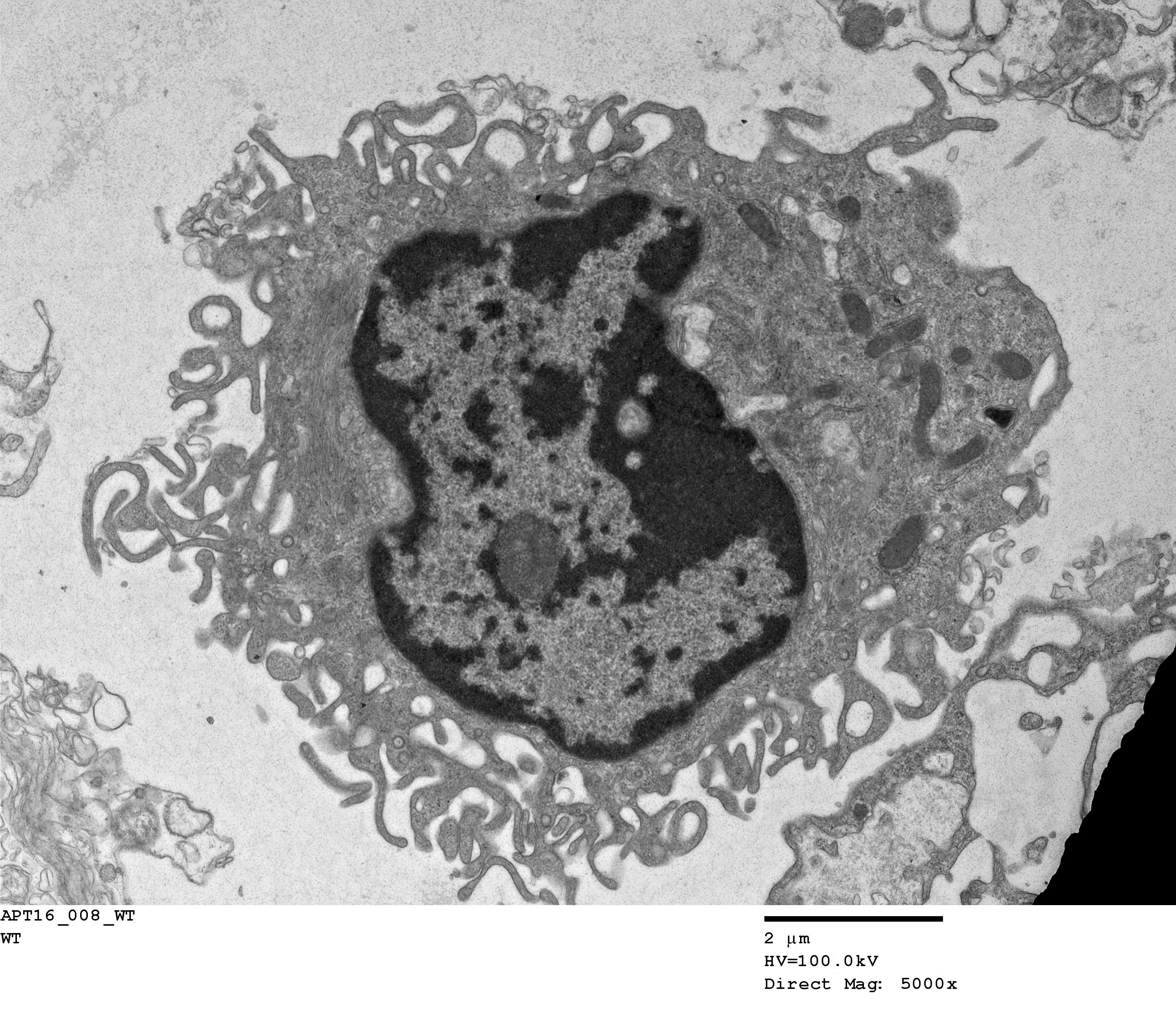

Supplement: Figure 3—source data 1. [file elife-66703-fig3-data1.zip › control EM Pt1 Fig3ABDE/APT16_008_WT.TIF]

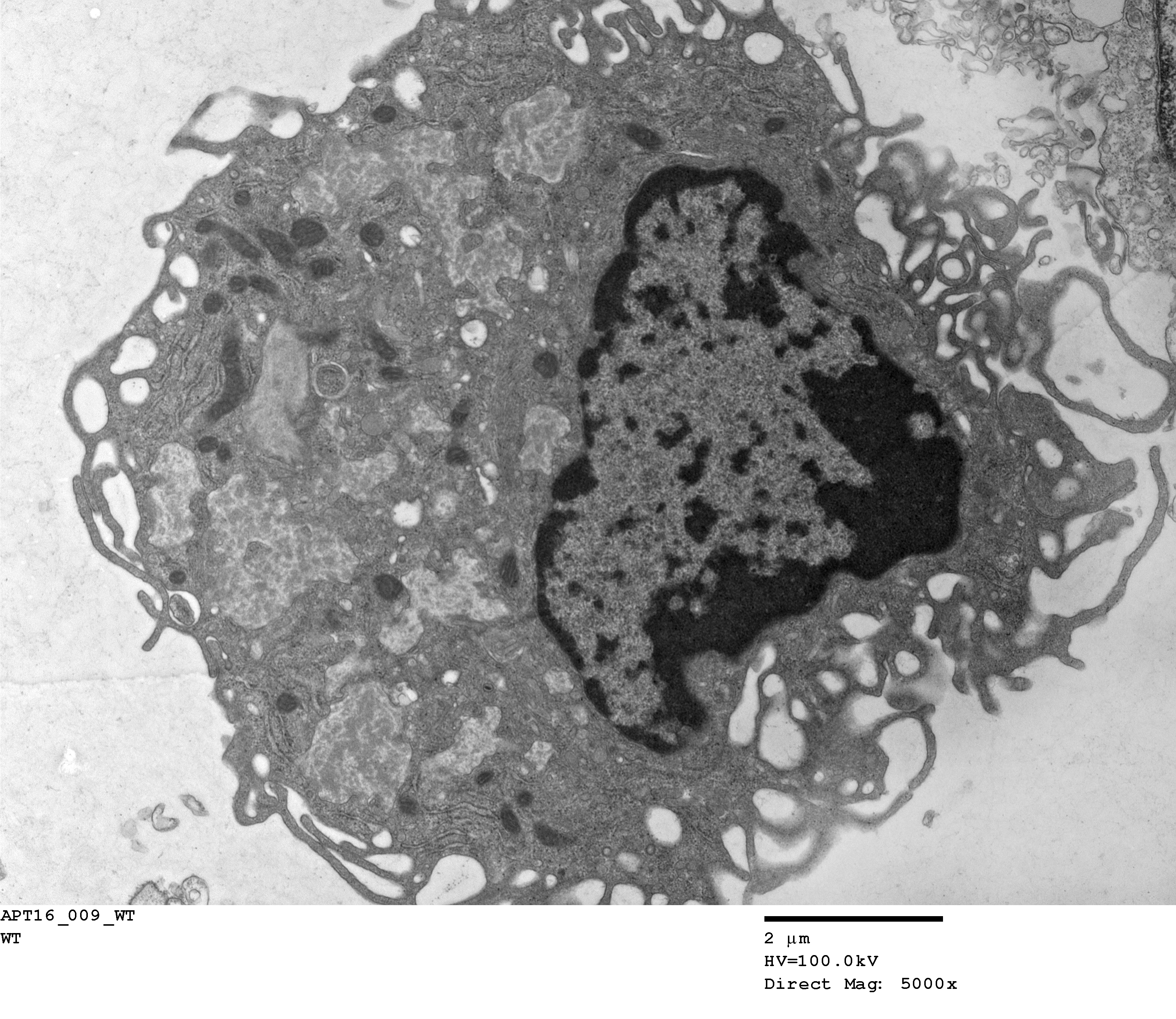

Supplement: Figure 3—source data 1. [file elife-66703-fig3-data1.zip › control EM Pt1 Fig3ABDE/APT16_009_WT.TIF]

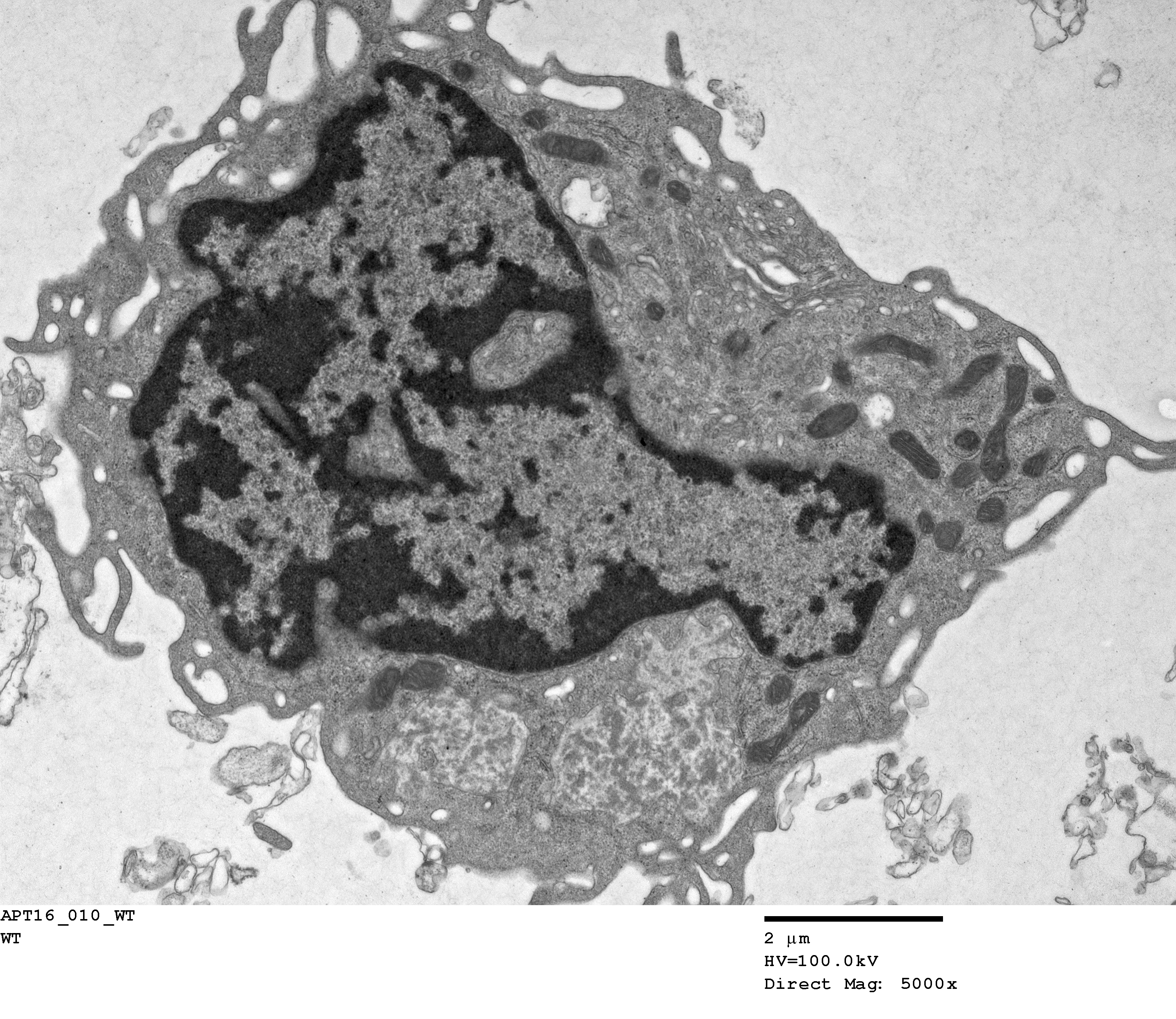

Supplement: Figure 3—source data 1. [file elife-66703-fig3-data1.zip › control EM Pt1 Fig3ABDE/APT16_010_WT.TIF]

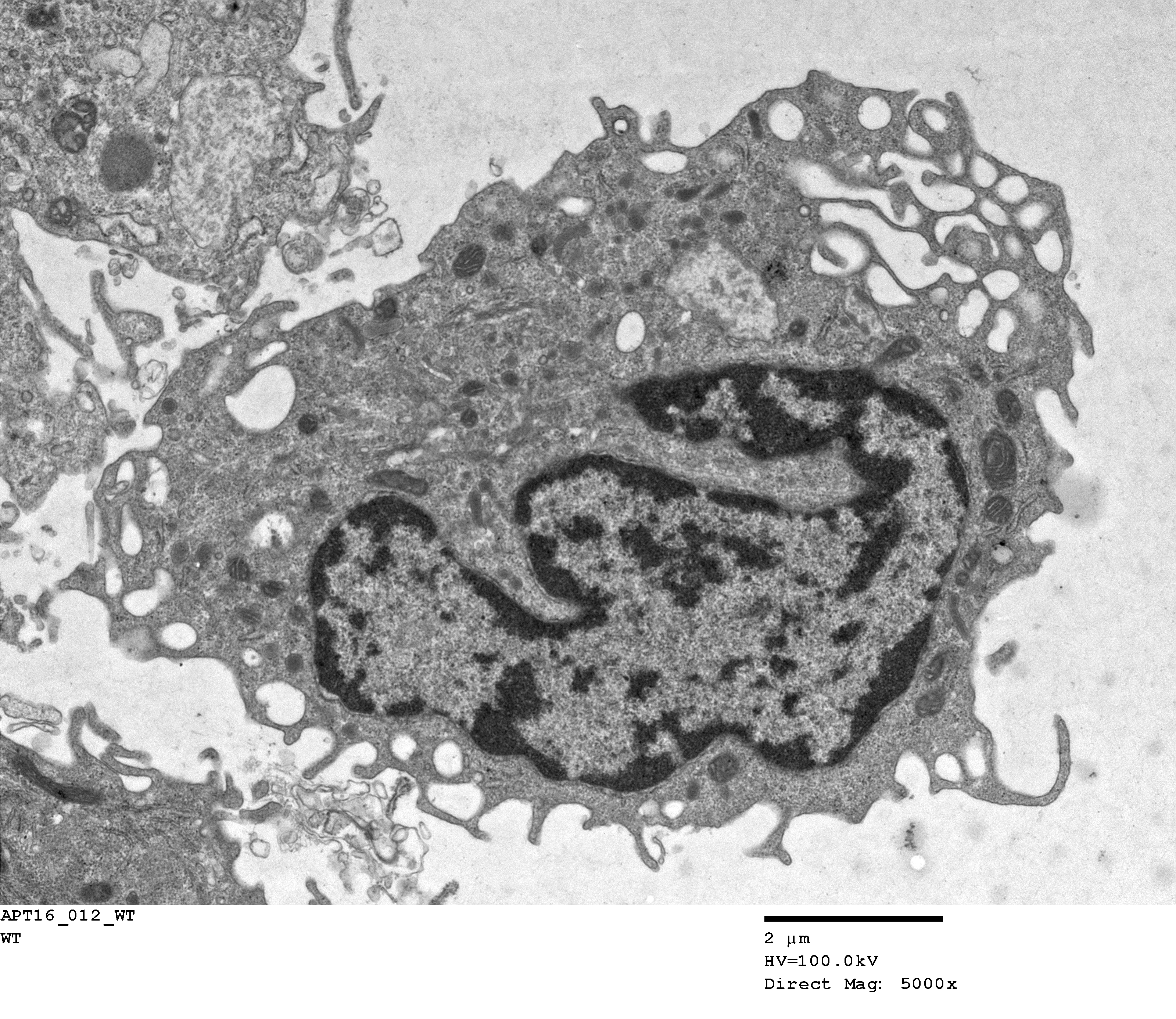

Supplement: Figure 3—source data 1. [file elife-66703-fig3-data1.zip › control EM Pt1 Fig3ABDE/APT16_012_WT.TIF]

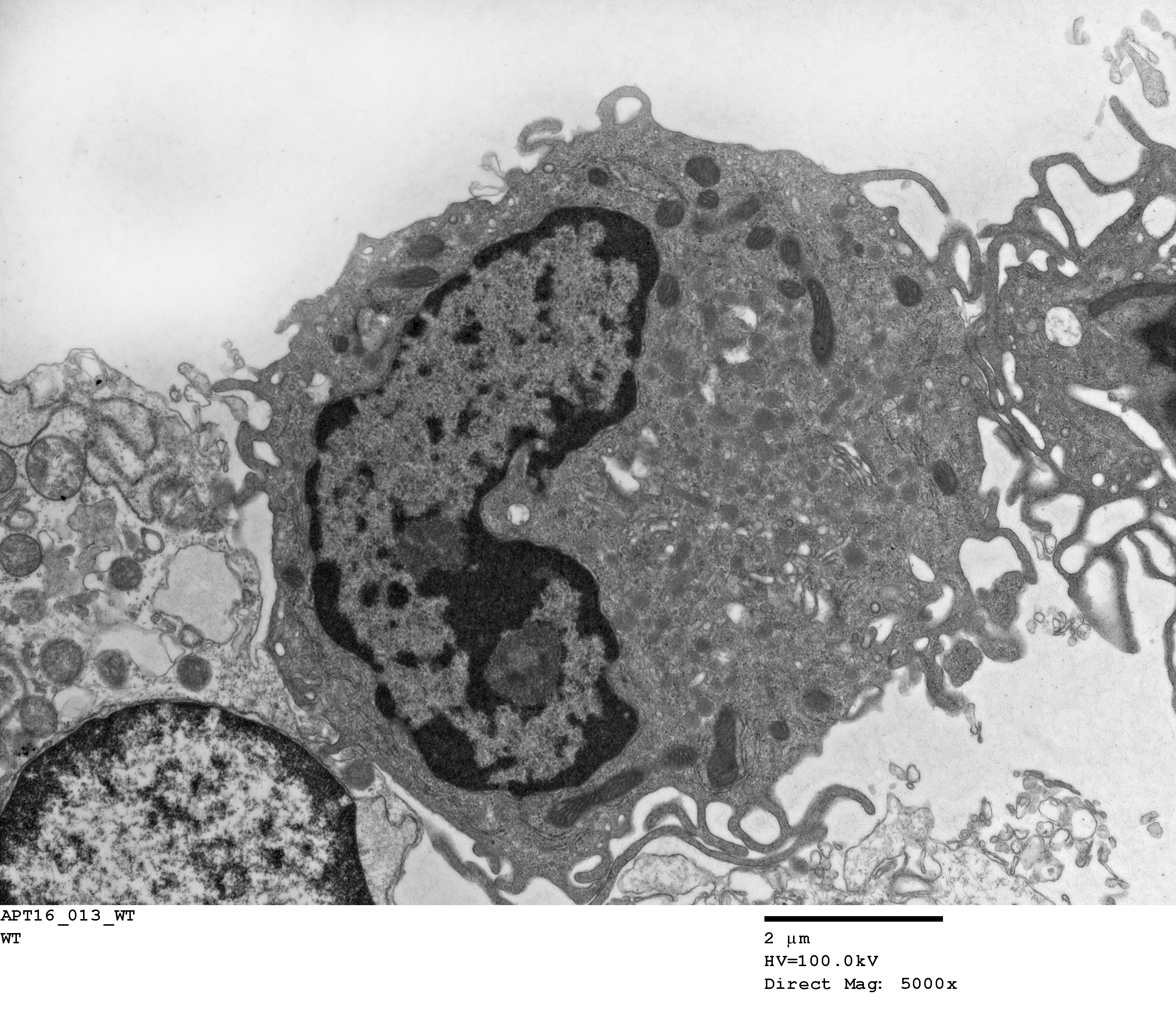

Supplement: Figure 3—source data 1. [file elife-66703-fig3-data1.zip › control EM Pt1 Fig3ABDE/APT16_013_WT.TIF]

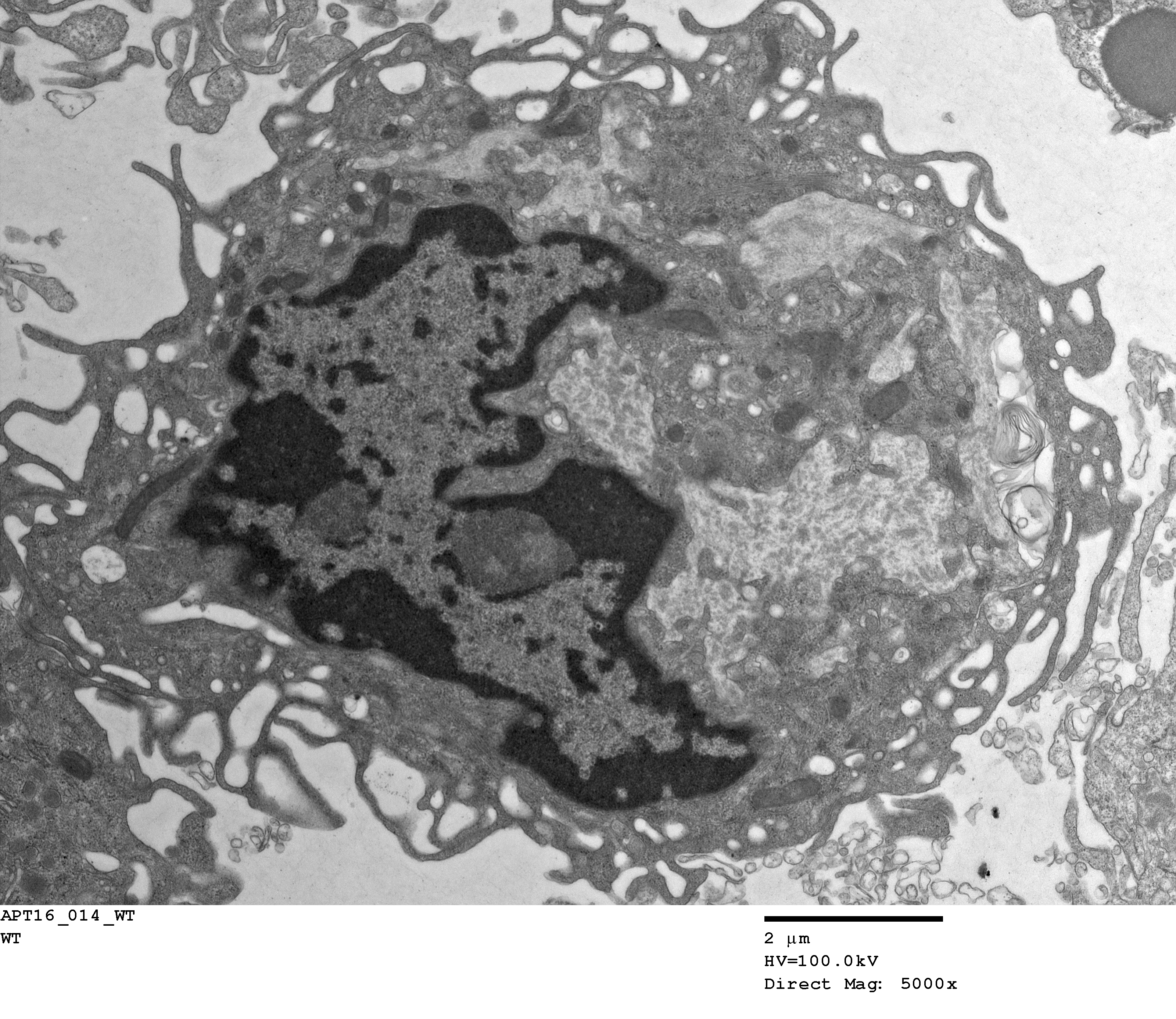

Supplement: Figure 3—source data 1. [file elife-66703-fig3-data1.zip › control EM Pt1 Fig3ABDE/APT16_014_WT.TIF]

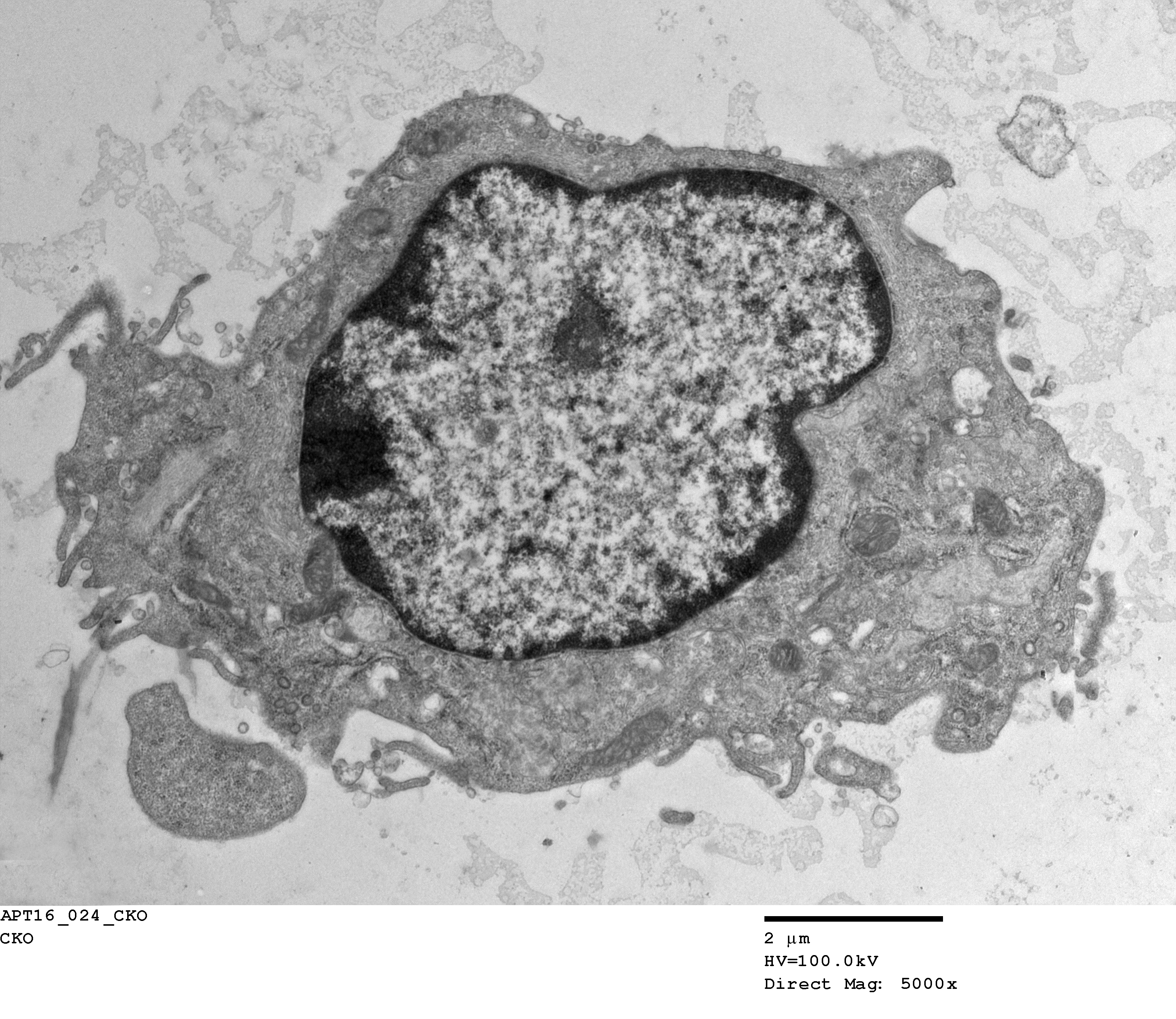

Supplement: Figure 3—source data 2. [file elife-66703-fig3-data2.zip › miR-146b CKO EM pt 1 Fig 3ABDE/APT16_024_CKO.TIF]

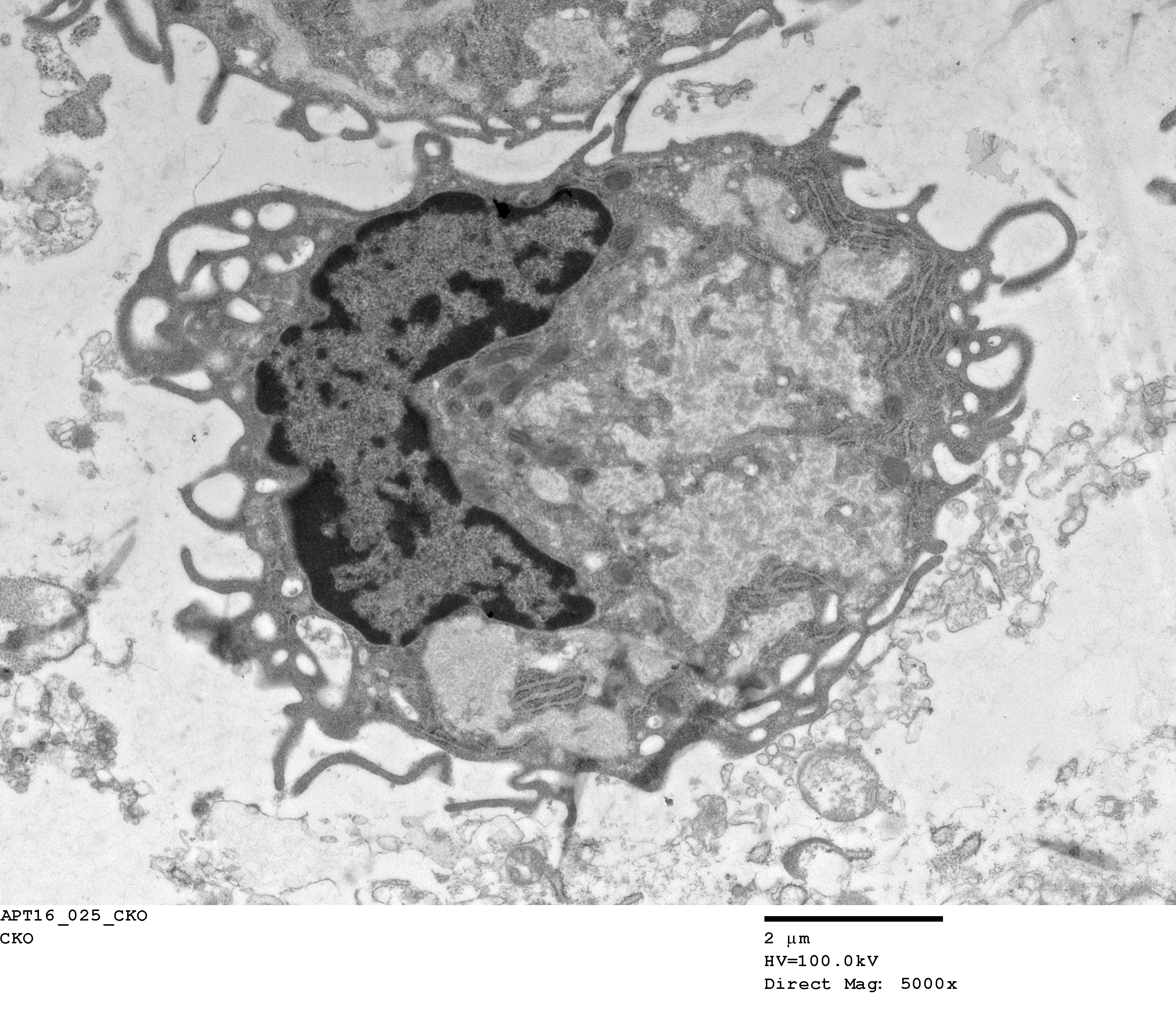

Supplement: Figure 3—source data 2. [file elife-66703-fig3-data2.zip › miR-146b CKO EM pt 1 Fig 3ABDE/APT16_025_CKO.TIF]

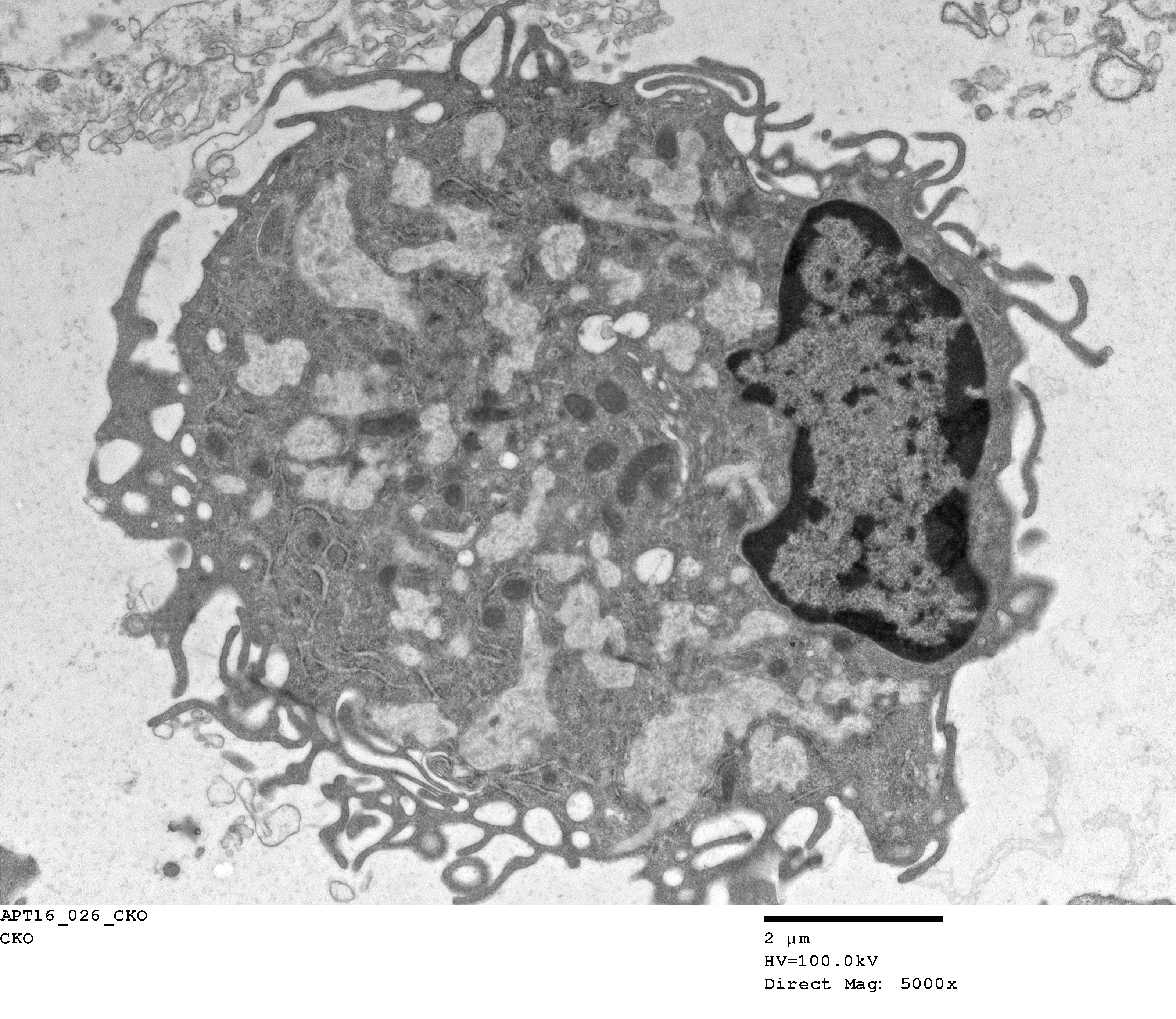

Supplement: Figure 3—source data 2. [file elife-66703-fig3-data2.zip › miR-146b CKO EM pt 1 Fig 3ABDE/APT16_026_CKO.TIF]

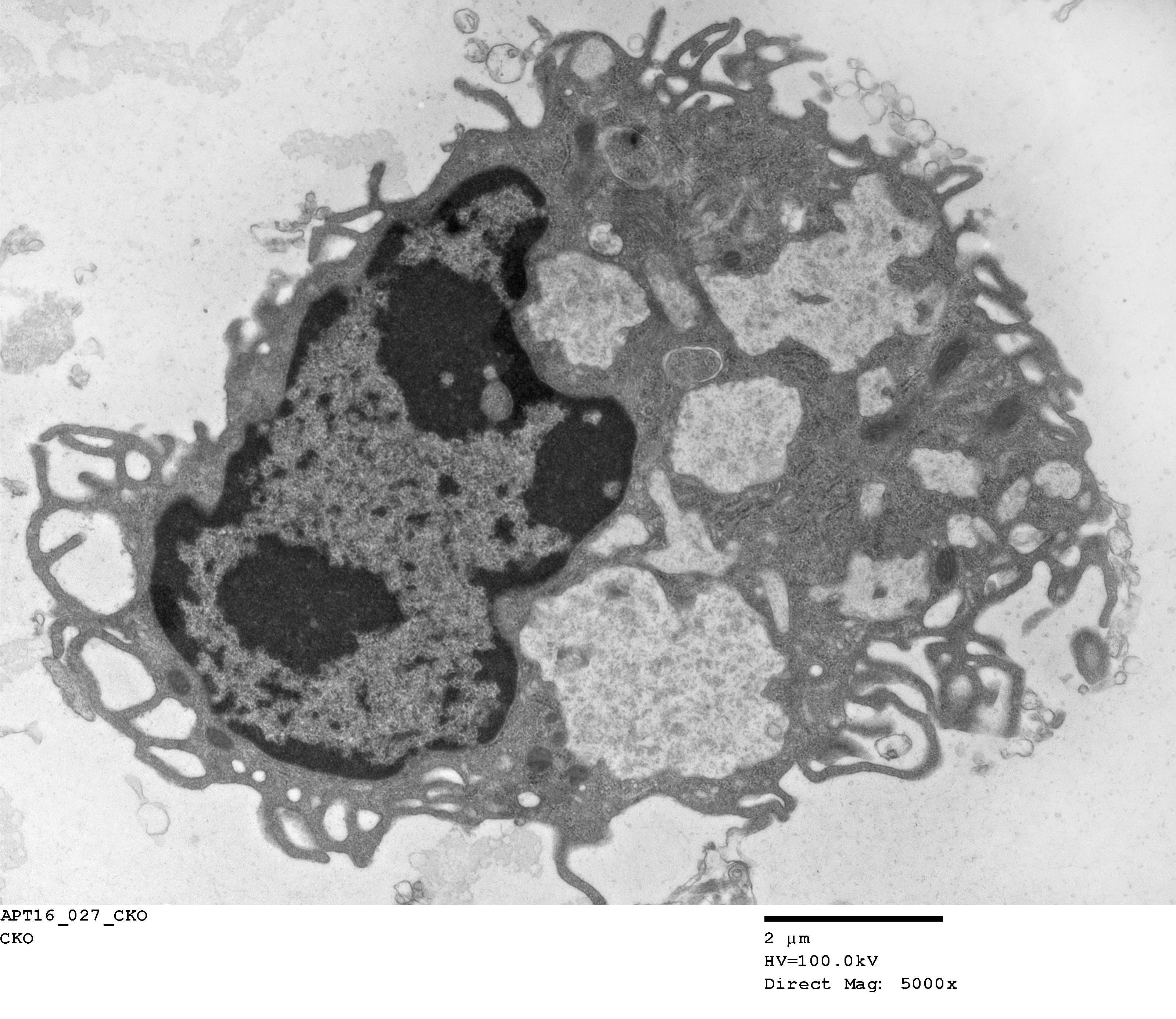

Supplement: Figure 3—source data 2. [file elife-66703-fig3-data2.zip › miR-146b CKO EM pt 1 Fig 3ABDE/APT16_027_CKO.TIF]

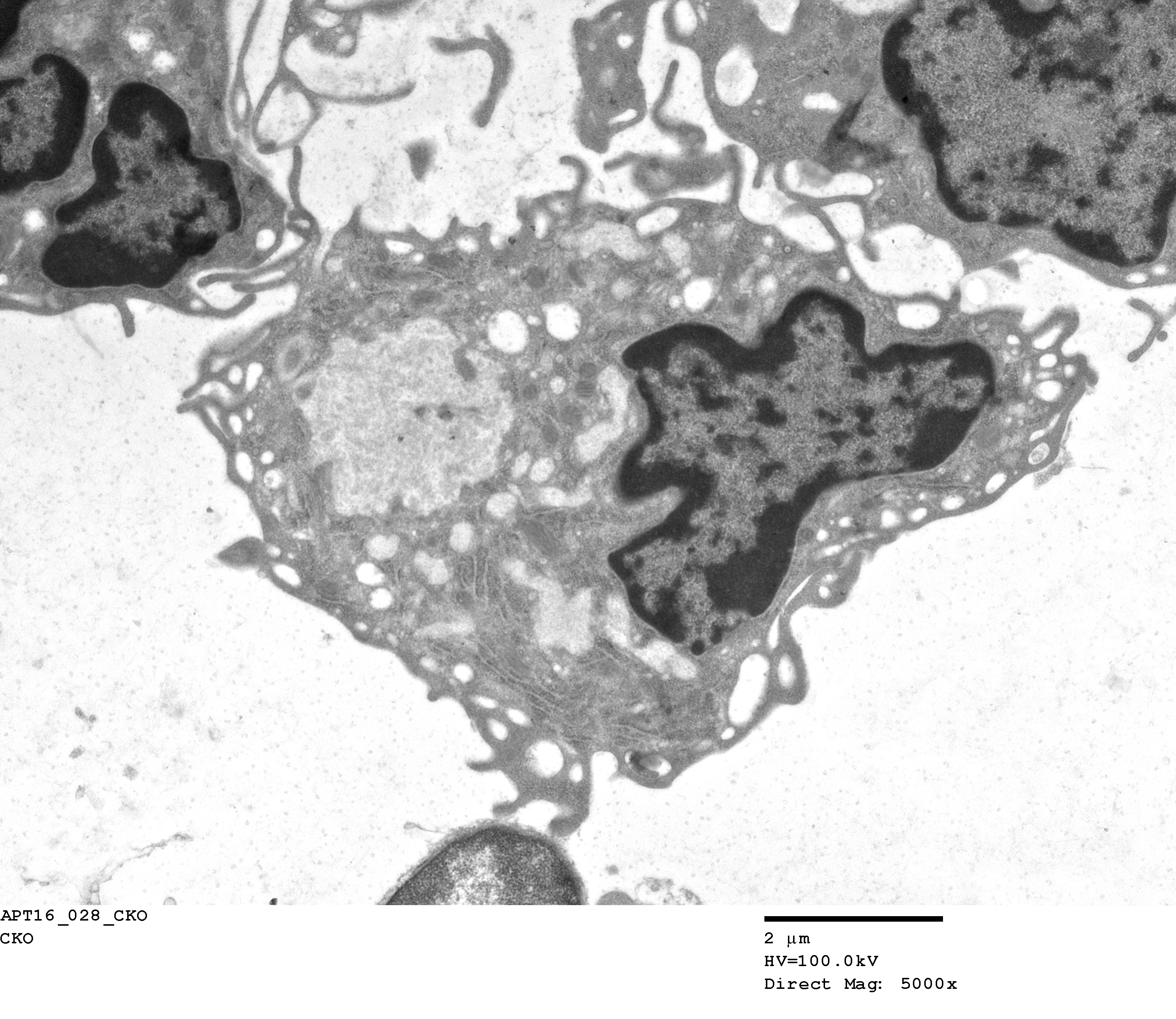

Supplement: Figure 3—source data 2. [file elife-66703-fig3-data2.zip › miR-146b CKO EM pt 1 Fig 3ABDE/APT16_028_CKO.TIF]

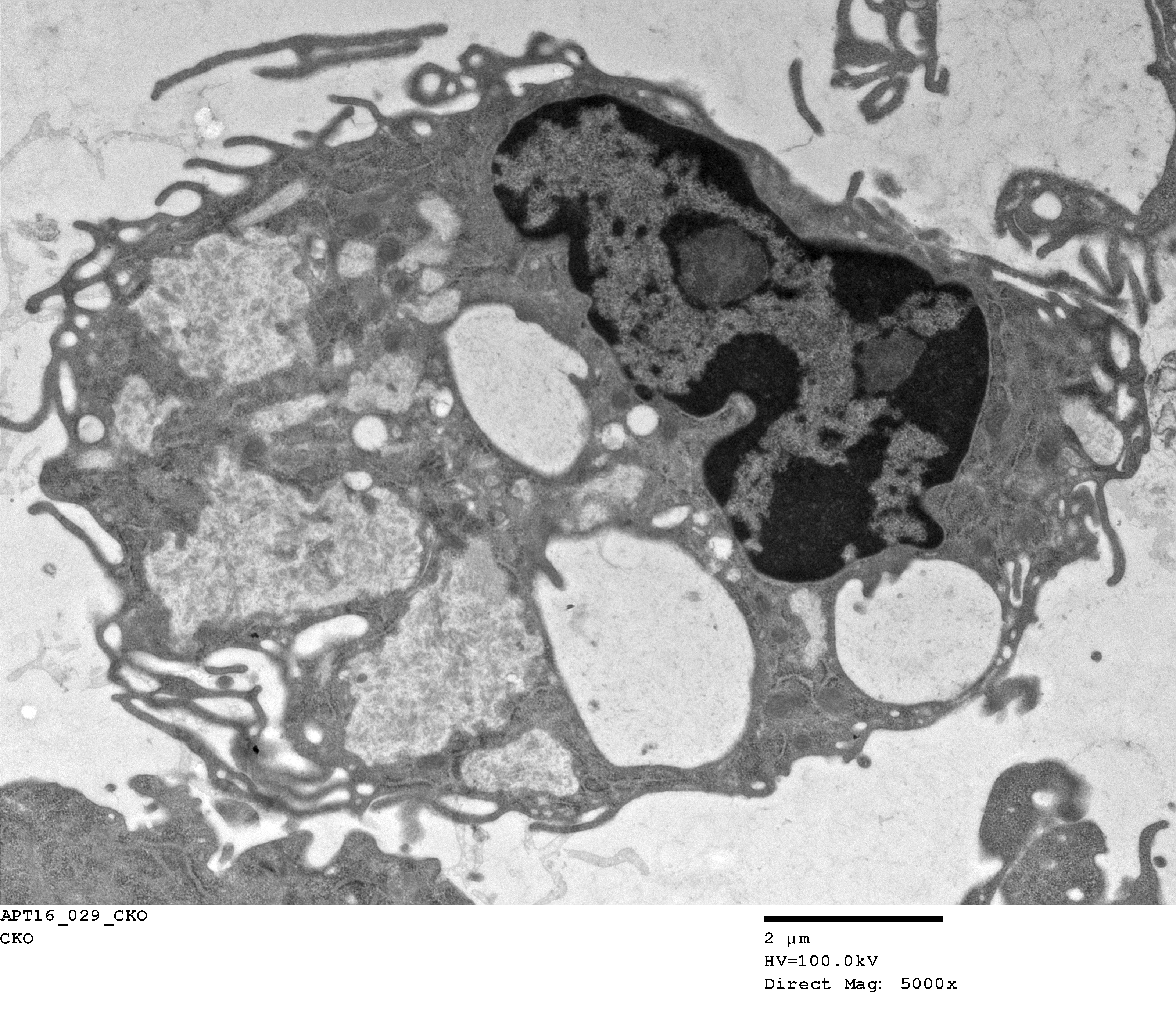

Supplement: Figure 3—source data 2. [file elife-66703-fig3-data2.zip › miR-146b CKO EM pt 1 Fig 3ABDE/APT16_029_CKO.TIF]

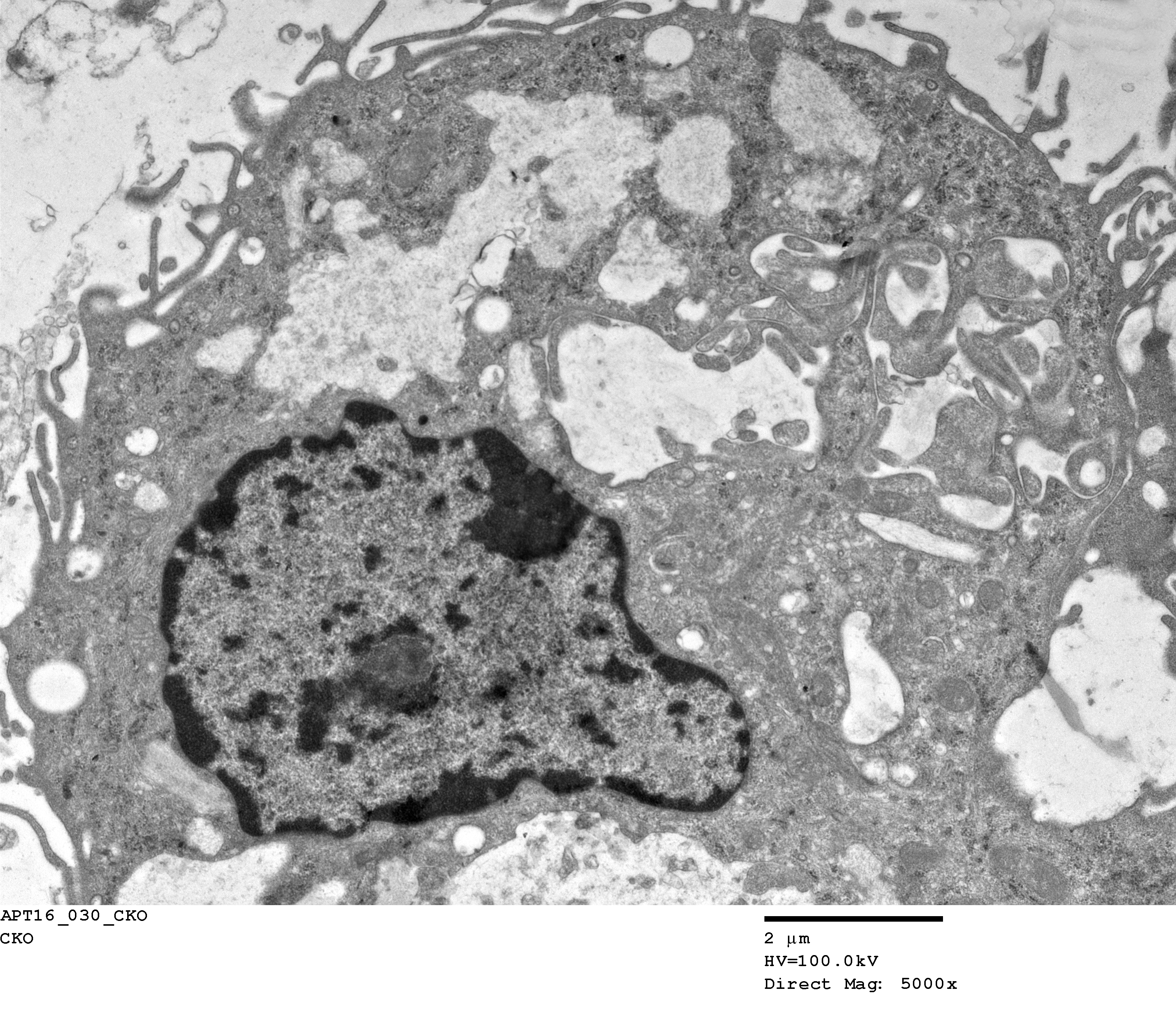

Supplement: Figure 3—source data 2. [file elife-66703-fig3-data2.zip › miR-146b CKO EM pt 1 Fig 3ABDE/APT16_030_CKO.TIF]

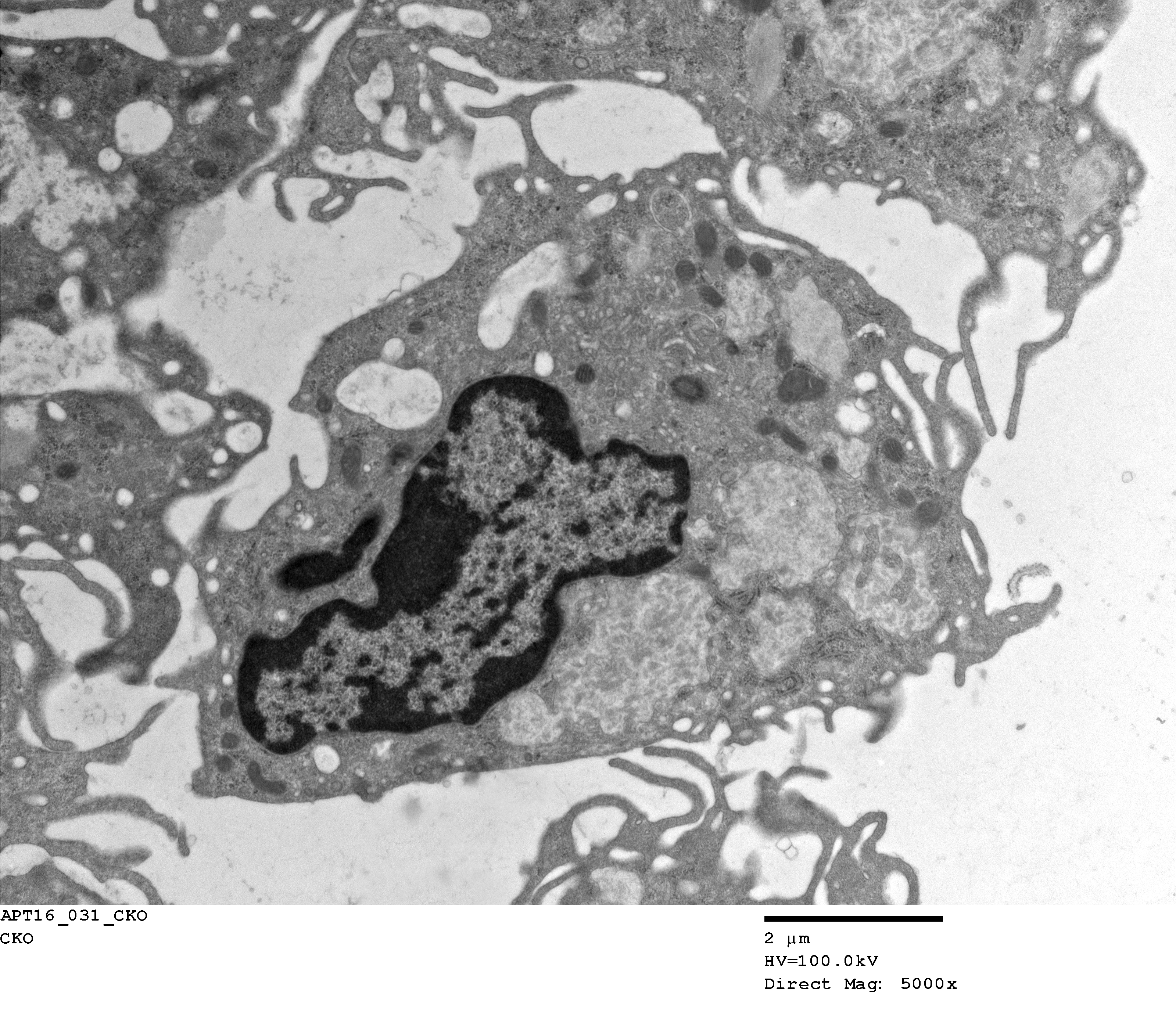

Supplement: Figure 3—source data 2. [file elife-66703-fig3-data2.zip › miR-146b CKO EM pt 1 Fig 3ABDE/APT16_031_CKO.TIF]

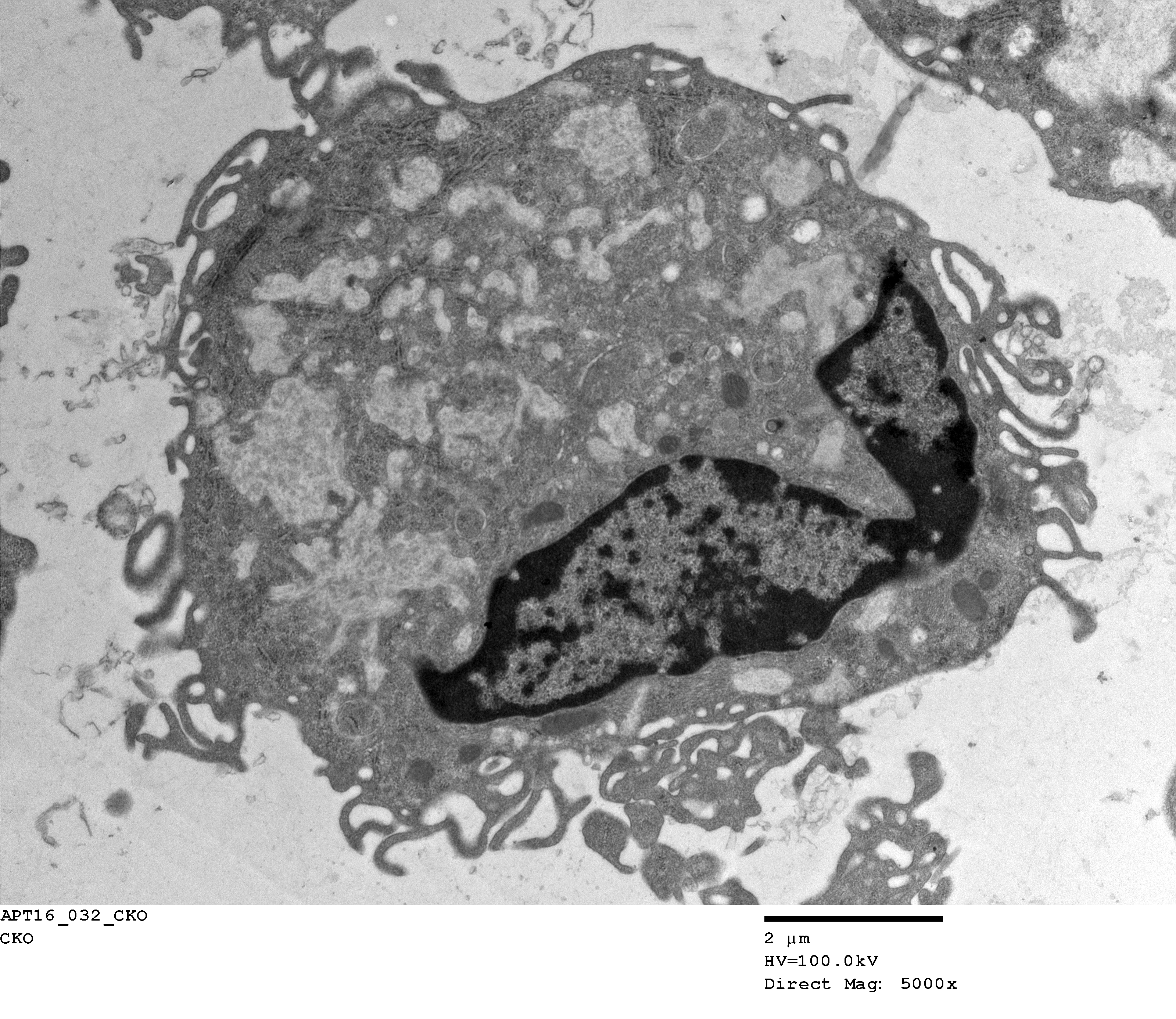

Supplement: Figure 3—source data 2. [file elife-66703-fig3-data2.zip › miR-146b CKO EM pt 1 Fig 3ABDE/APT16_032_CKO.TIF]

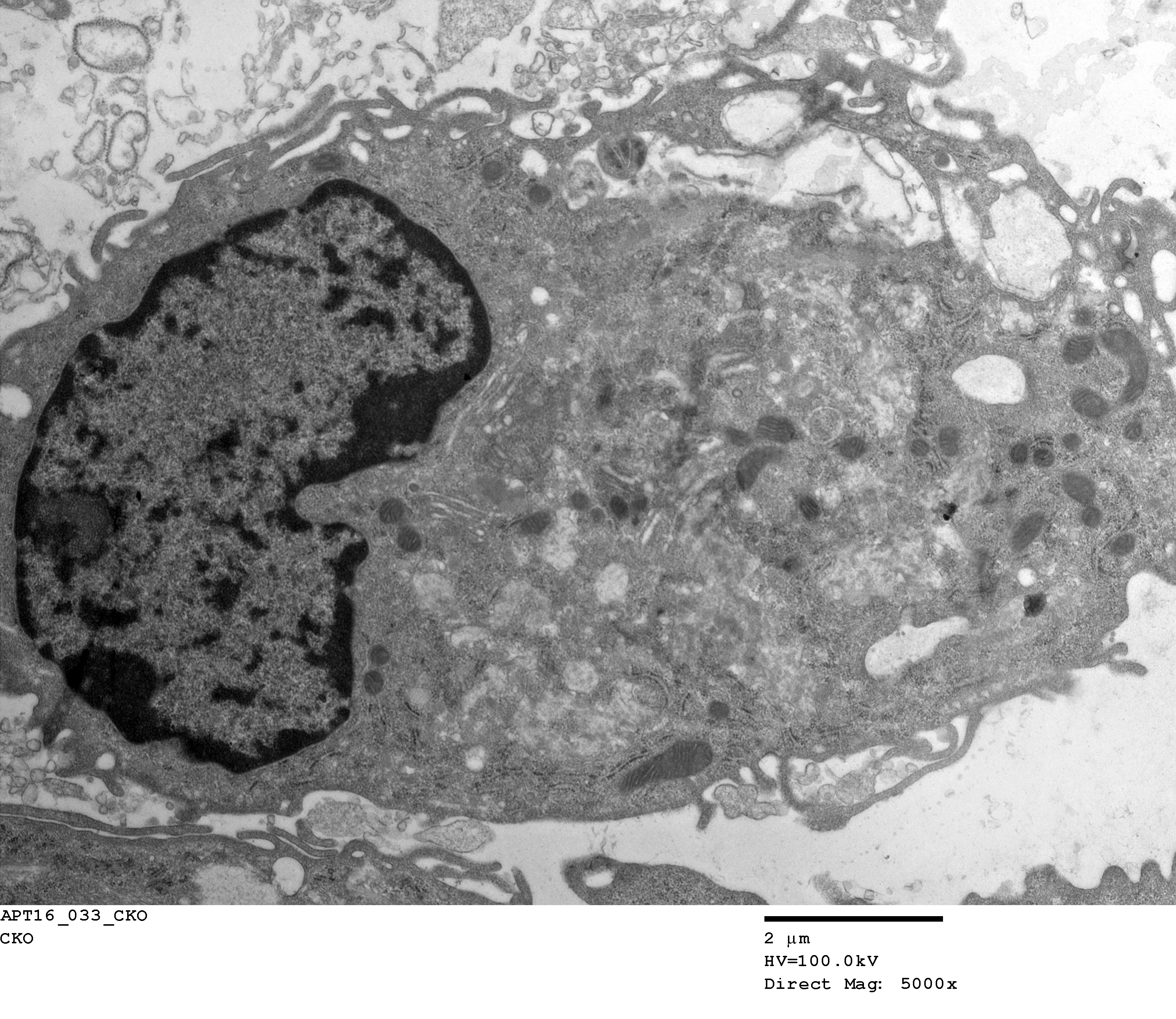

Supplement: Figure 3—source data 2. [file elife-66703-fig3-data2.zip › miR-146b CKO EM pt 1 Fig 3ABDE/APT16_033_CKO.TIF]

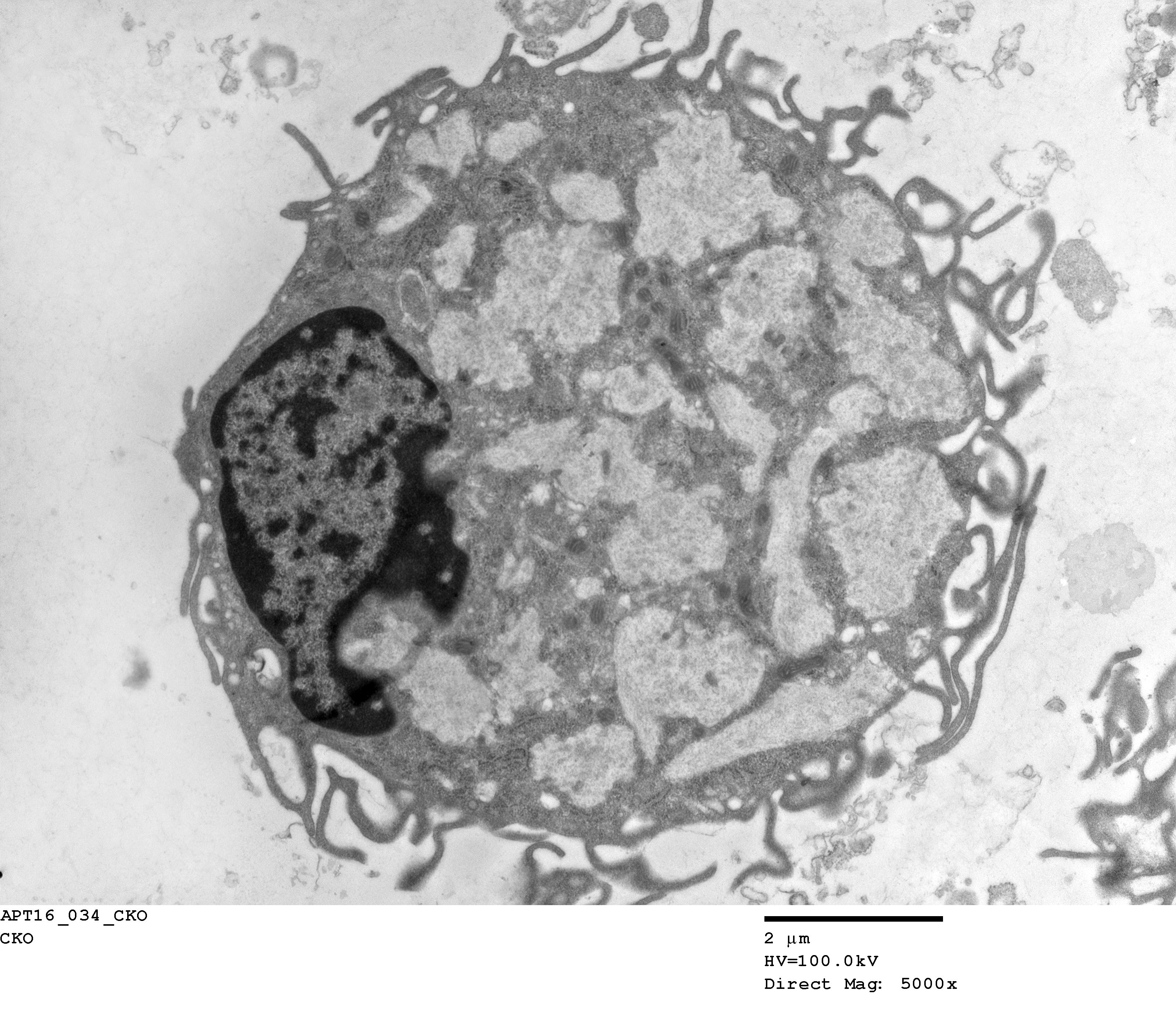

Supplement: Figure 3—source data 2. [file elife-66703-fig3-data2.zip › miR-146b CKO EM pt 1 Fig 3ABDE/APT16_034_CKO.TIF]

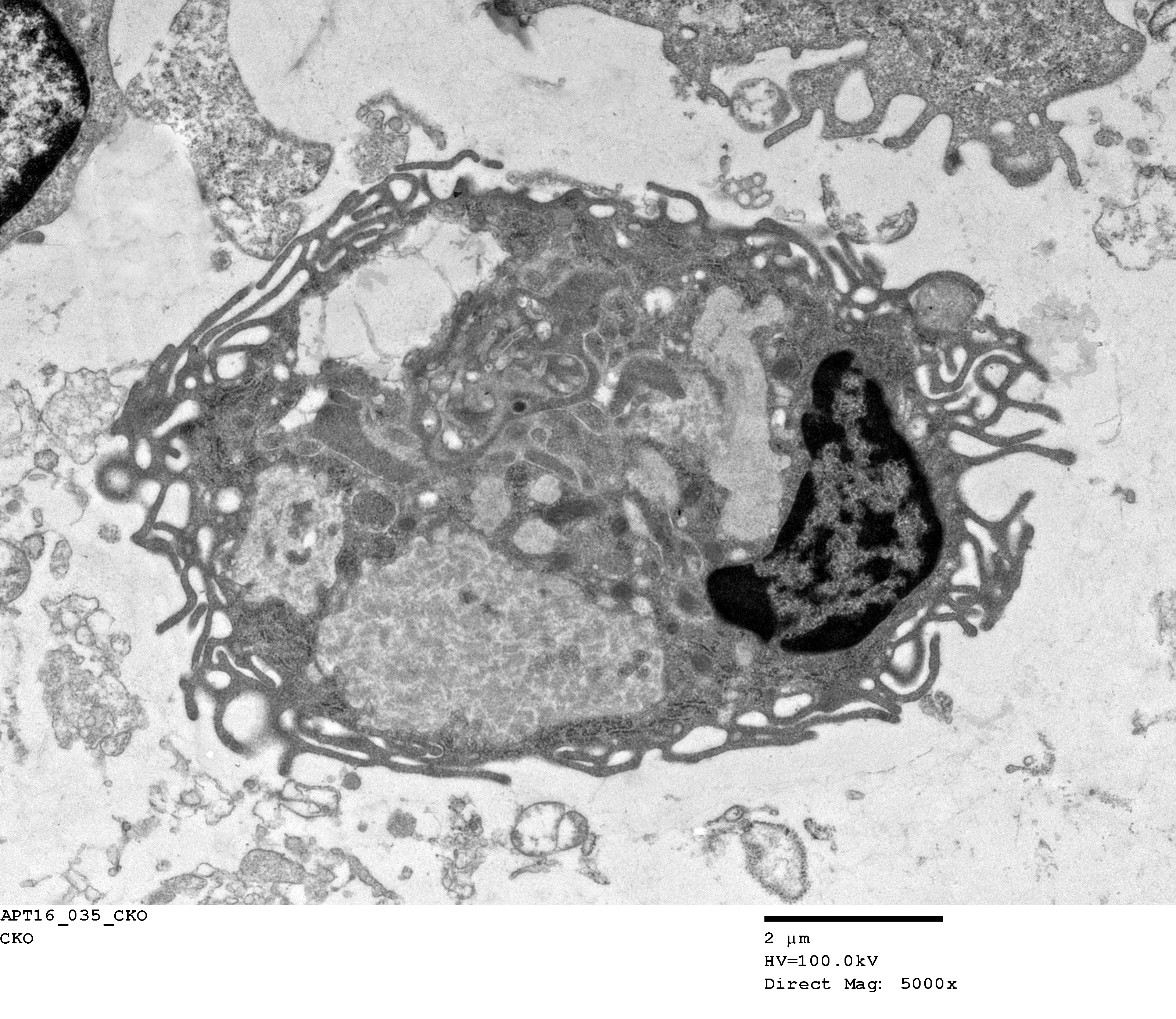

Supplement: Figure 3—source data 2. [file elife-66703-fig3-data2.zip › miR-146b CKO EM pt 1 Fig 3ABDE/APT16_035_CKO.TIF]

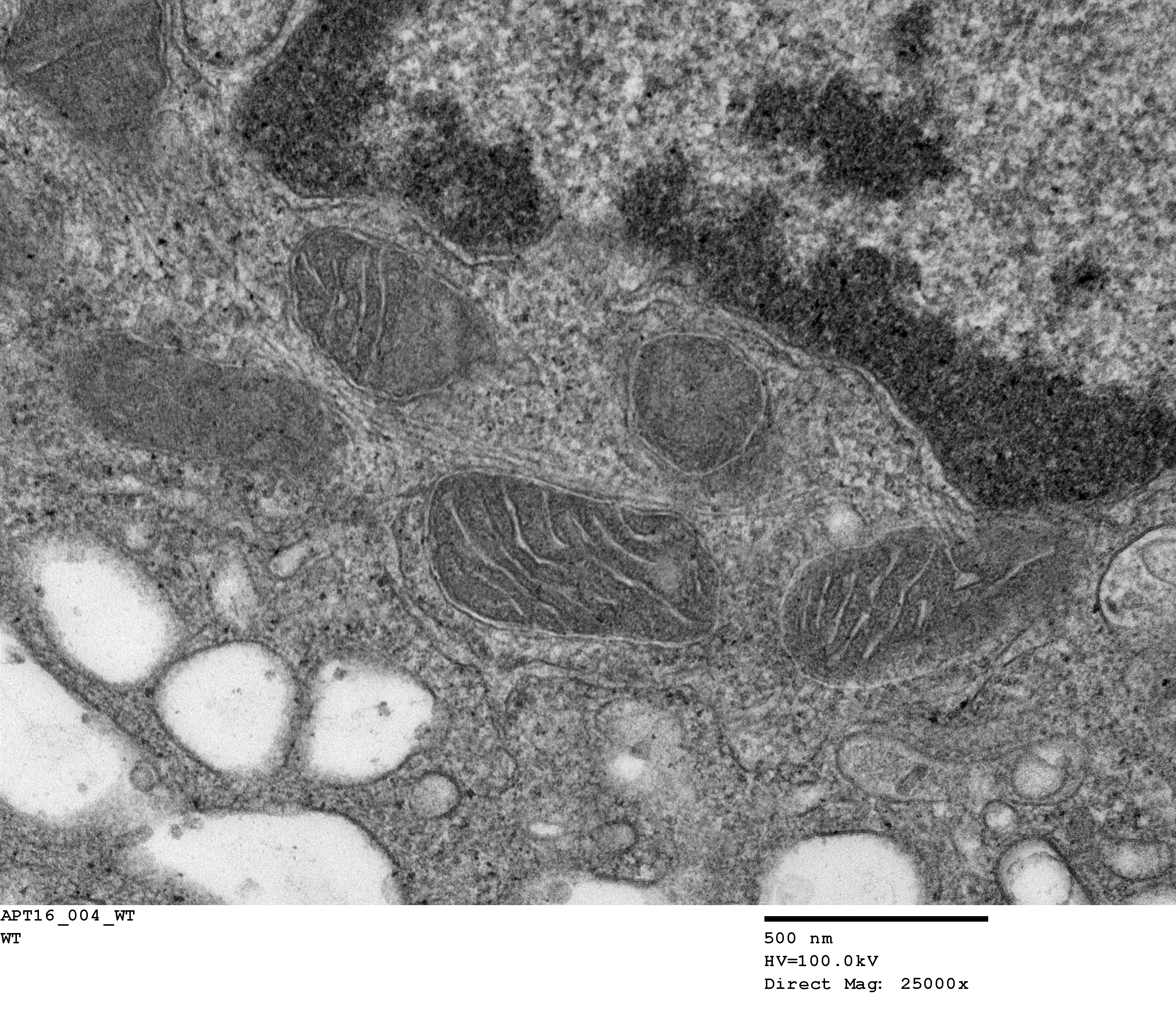

Supplement: Figure 3—source data 4. [file elife-66703-fig3-data4.zip › control EM Pt 2 Fig 3ABDE/APT16_004_WT.TIF]

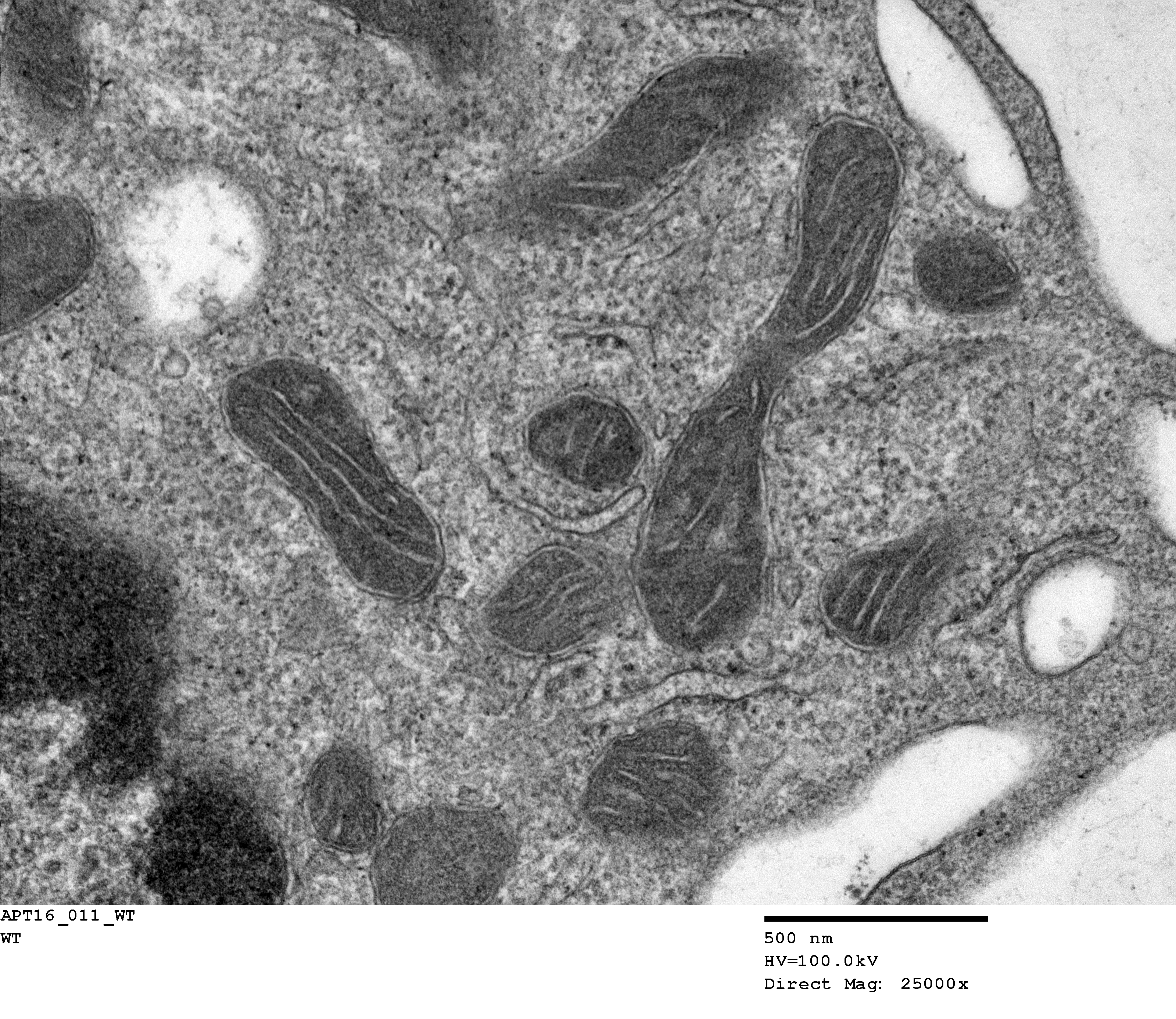

Supplement: Figure 3—source data 4. [file elife-66703-fig3-data4.zip › control EM Pt 2 Fig 3ABDE/APT16_011_WT.TIF]

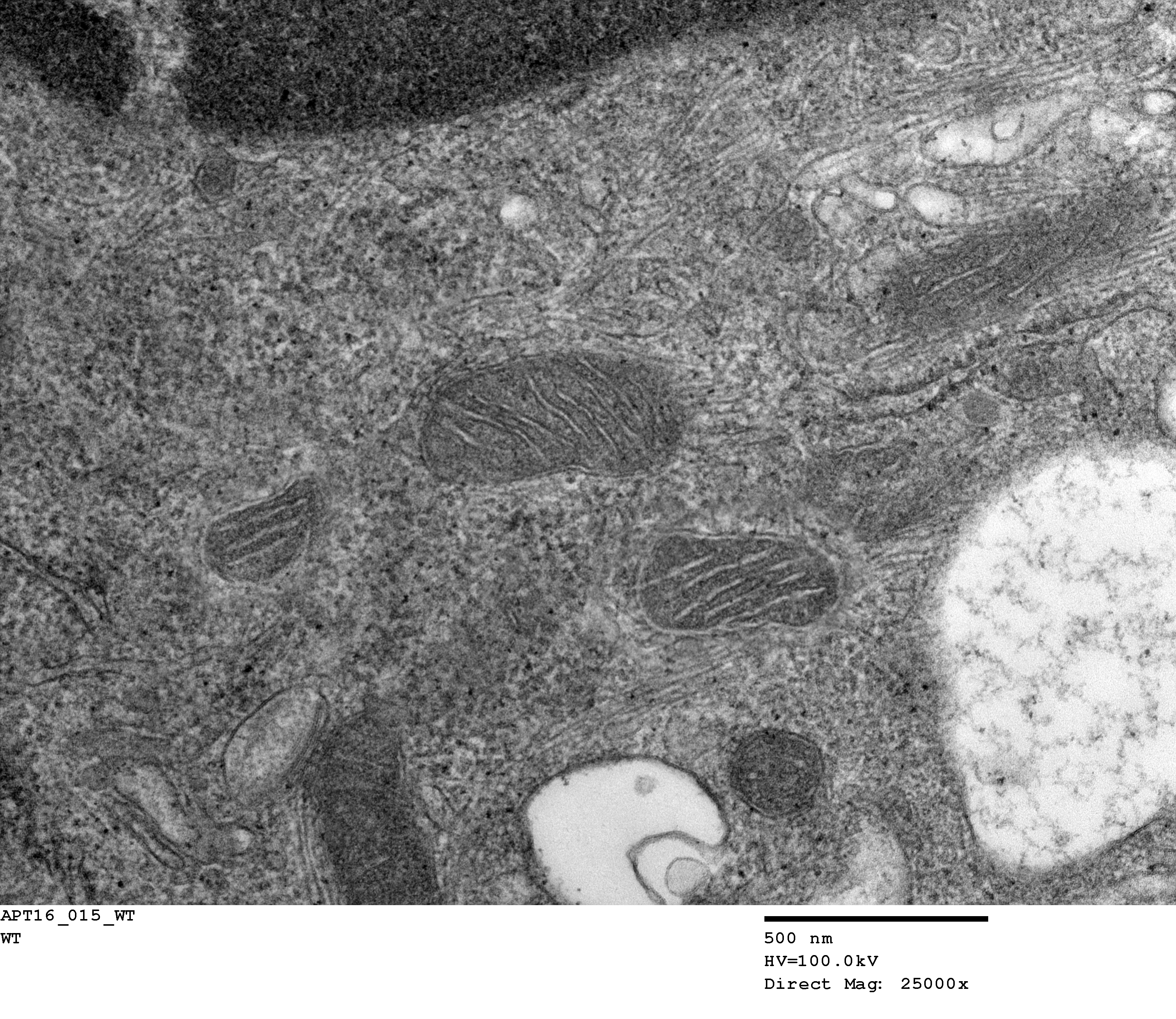

Supplement: Figure 3—source data 4. [file elife-66703-fig3-data4.zip › control EM Pt 2 Fig 3ABDE/APT16_015_WT.TIF]

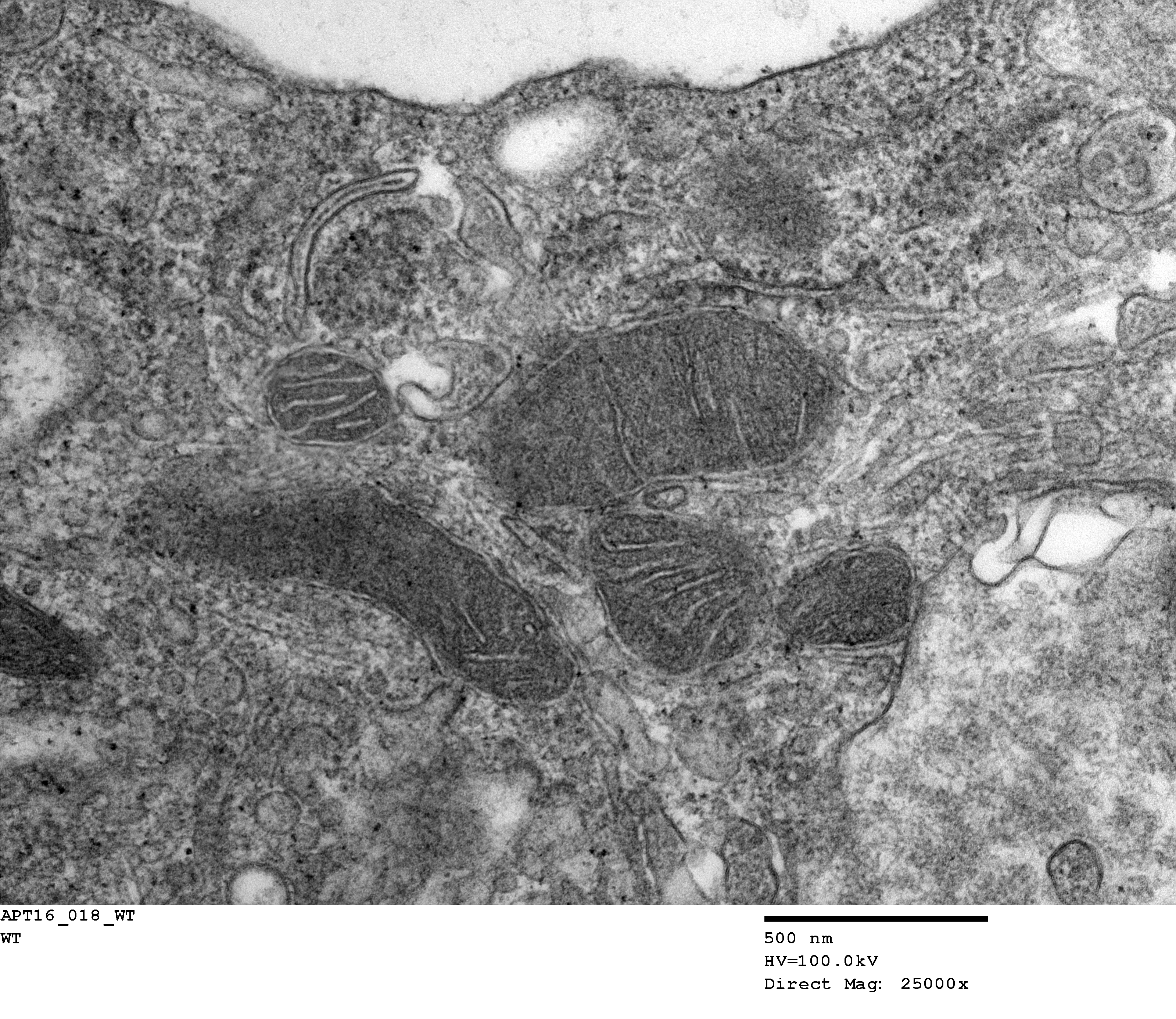

Supplement: Figure 3—source data 4. [file elife-66703-fig3-data4.zip › control EM Pt 2 Fig 3ABDE/APT16_018_WT.TIF]

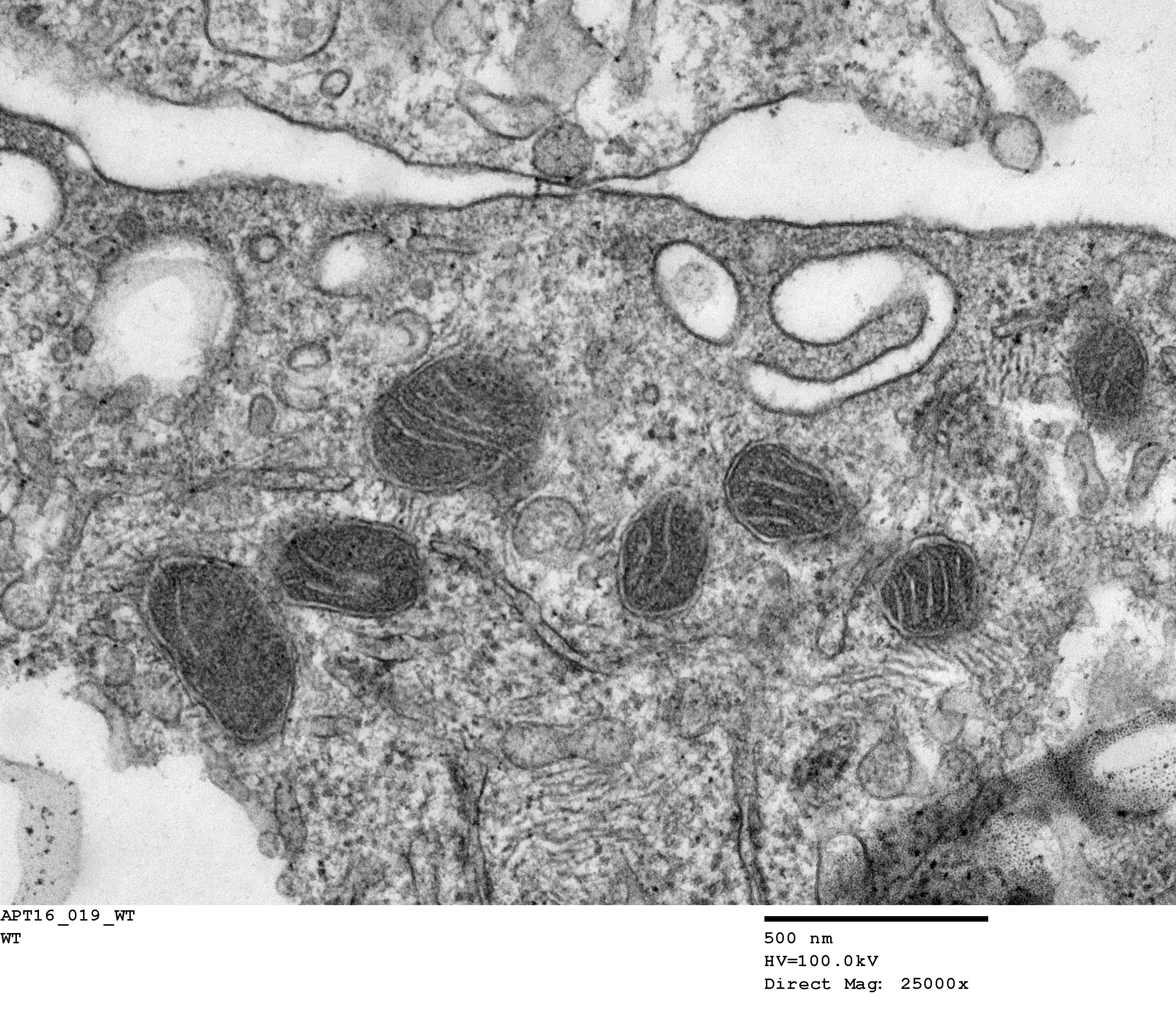

Supplement: Figure 3—source data 4. [file elife-66703-fig3-data4.zip › control EM Pt 2 Fig 3ABDE/APT16_019_WT.TIF]

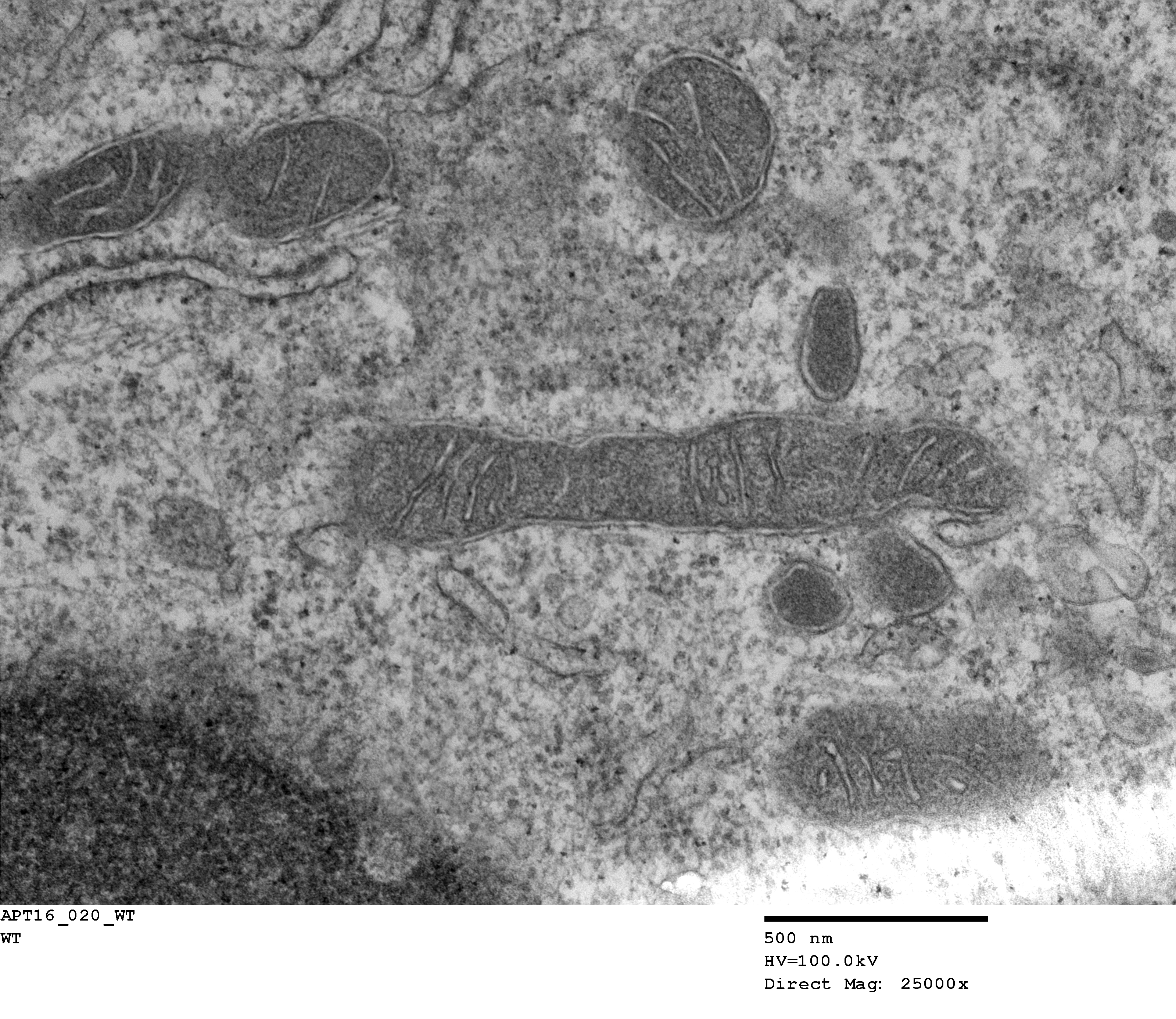

Supplement: Figure 3—source data 4. [file elife-66703-fig3-data4.zip › control EM Pt 2 Fig 3ABDE/APT16_020_WT.TIF]

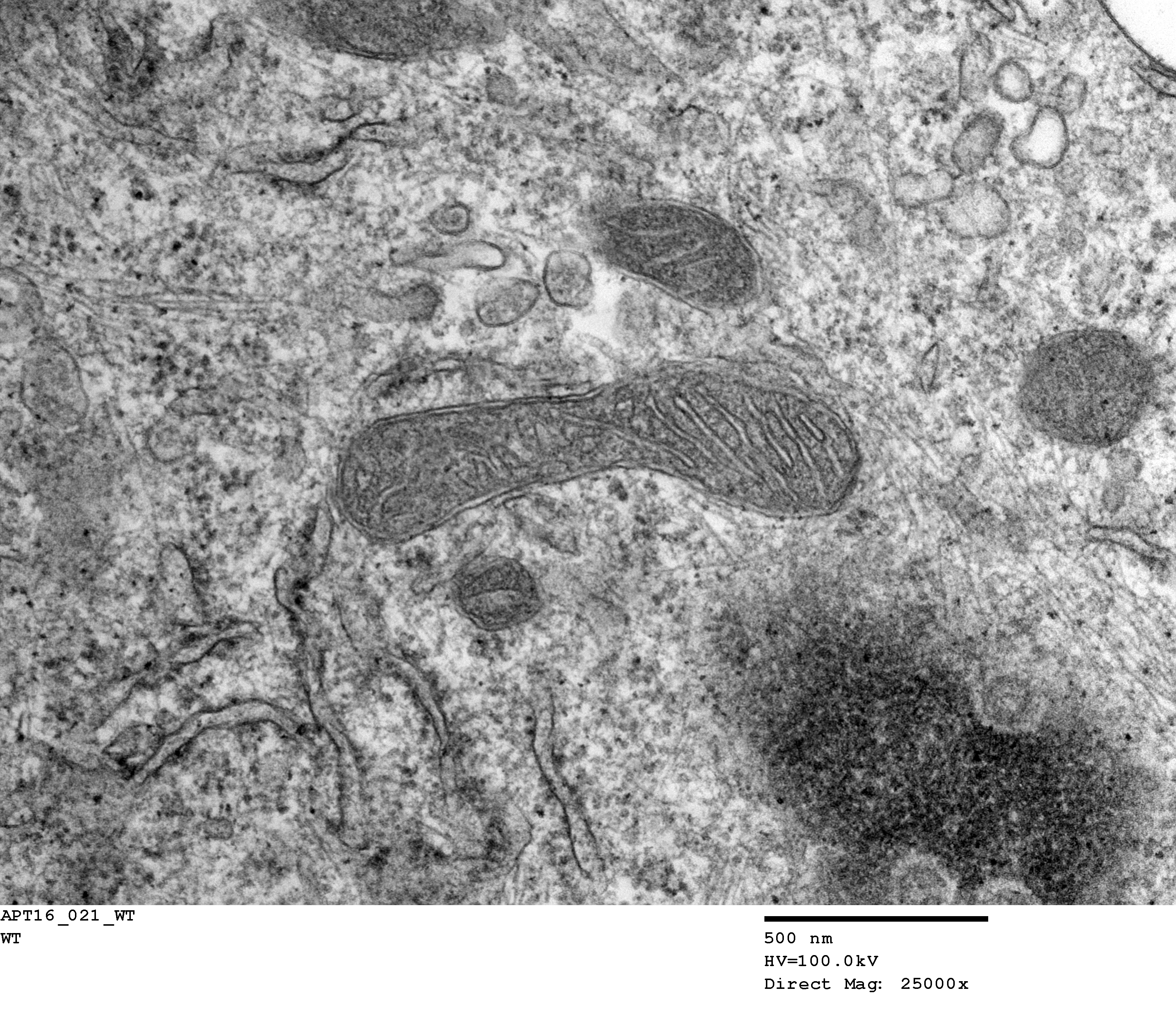

Supplement: Figure 3—source data 4. [file elife-66703-fig3-data4.zip › control EM Pt 2 Fig 3ABDE/APT16_021_WT.TIF]

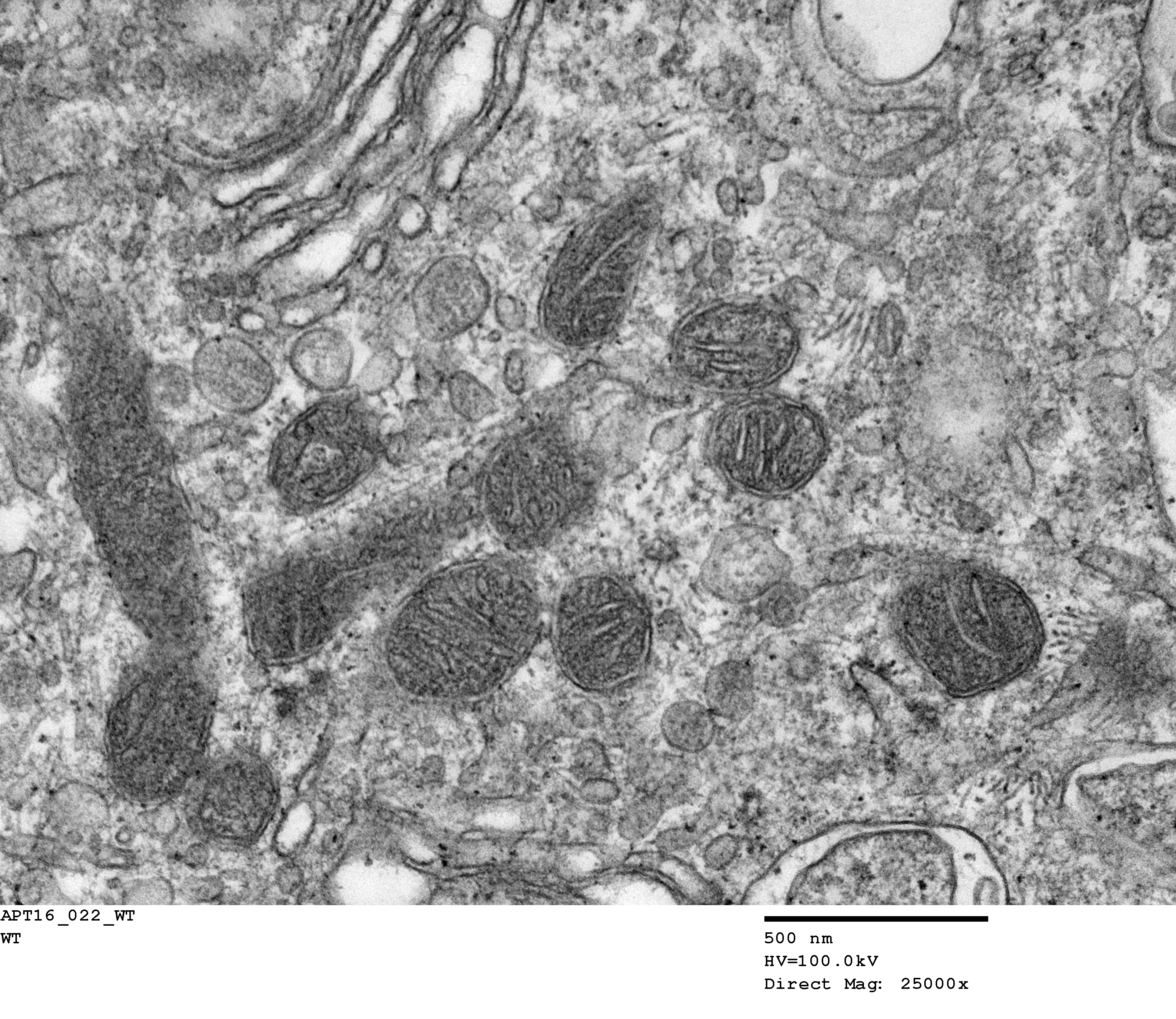

Supplement: Figure 3—source data 4. [file elife-66703-fig3-data4.zip › control EM Pt 2 Fig 3ABDE/APT16_022_WT.TIF]

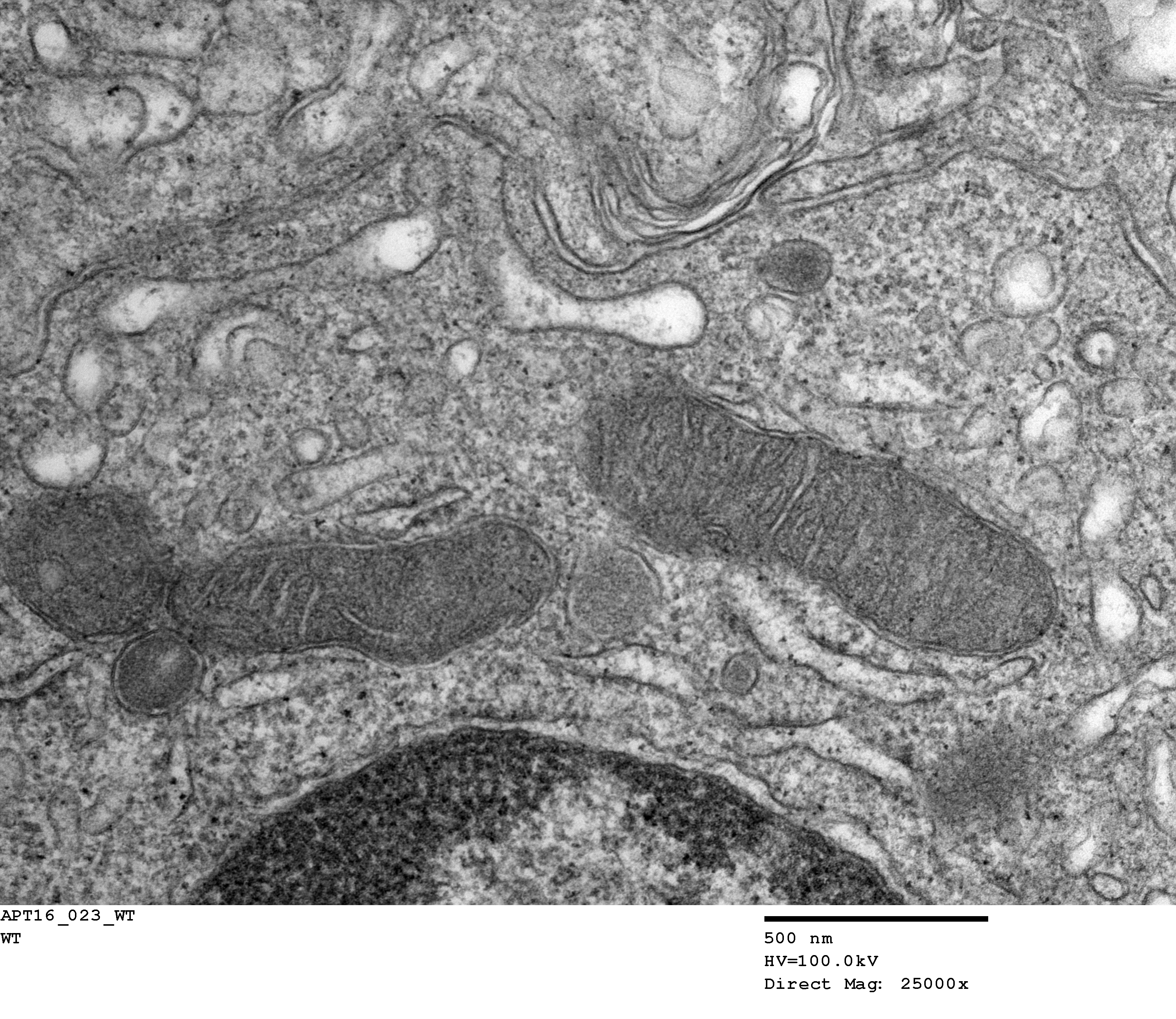

Supplement: Figure 3—source data 4. [file elife-66703-fig3-data4.zip › control EM Pt 2 Fig 3ABDE/APT16_023_WT.TIF]

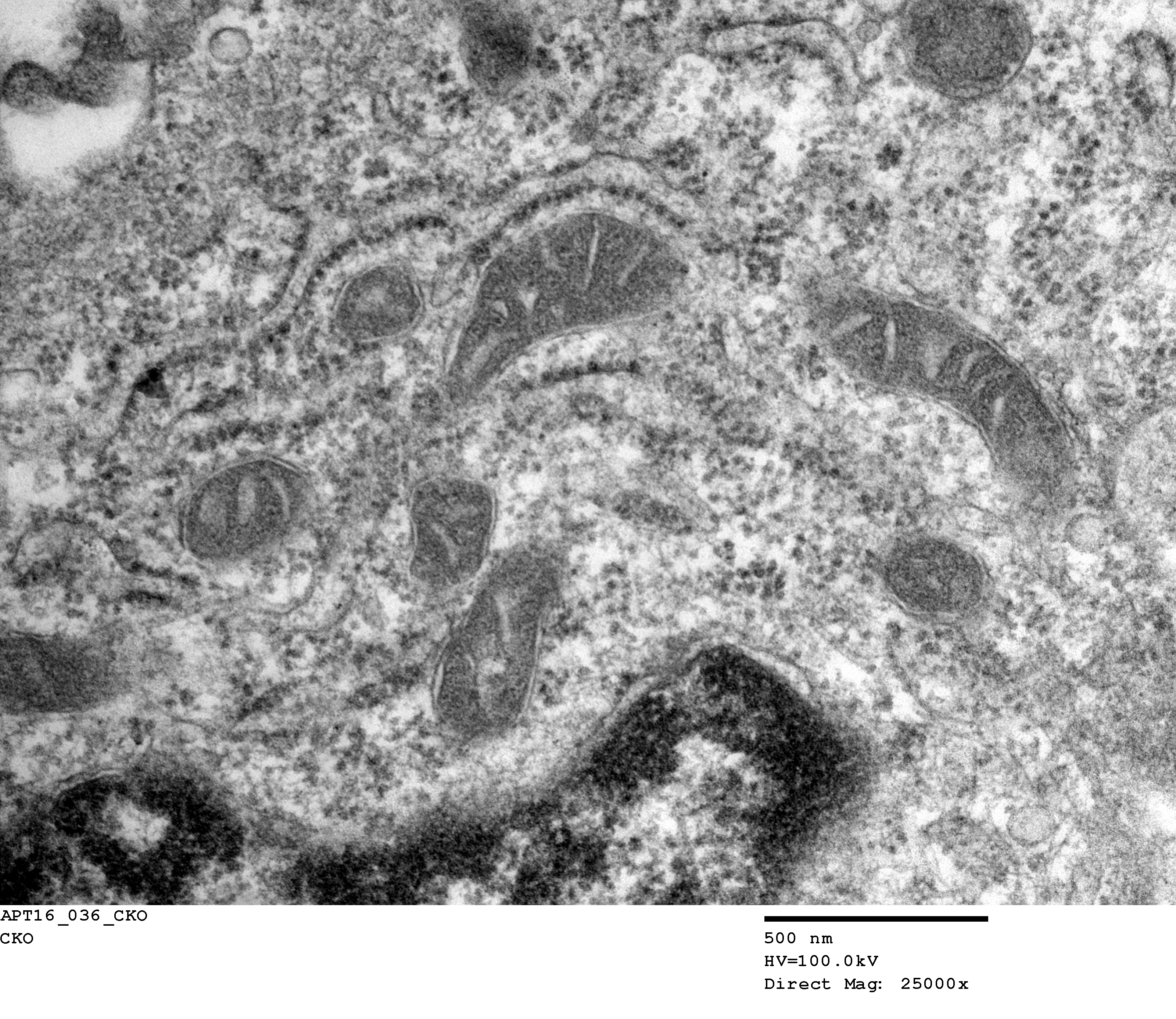

Supplement: Figure 3—source data 5. [file elife-66703-fig3-data5.zip › miR-146b CKO EM Pt 2 Fig 3ABDE/APT16_036_CKO.TIF]

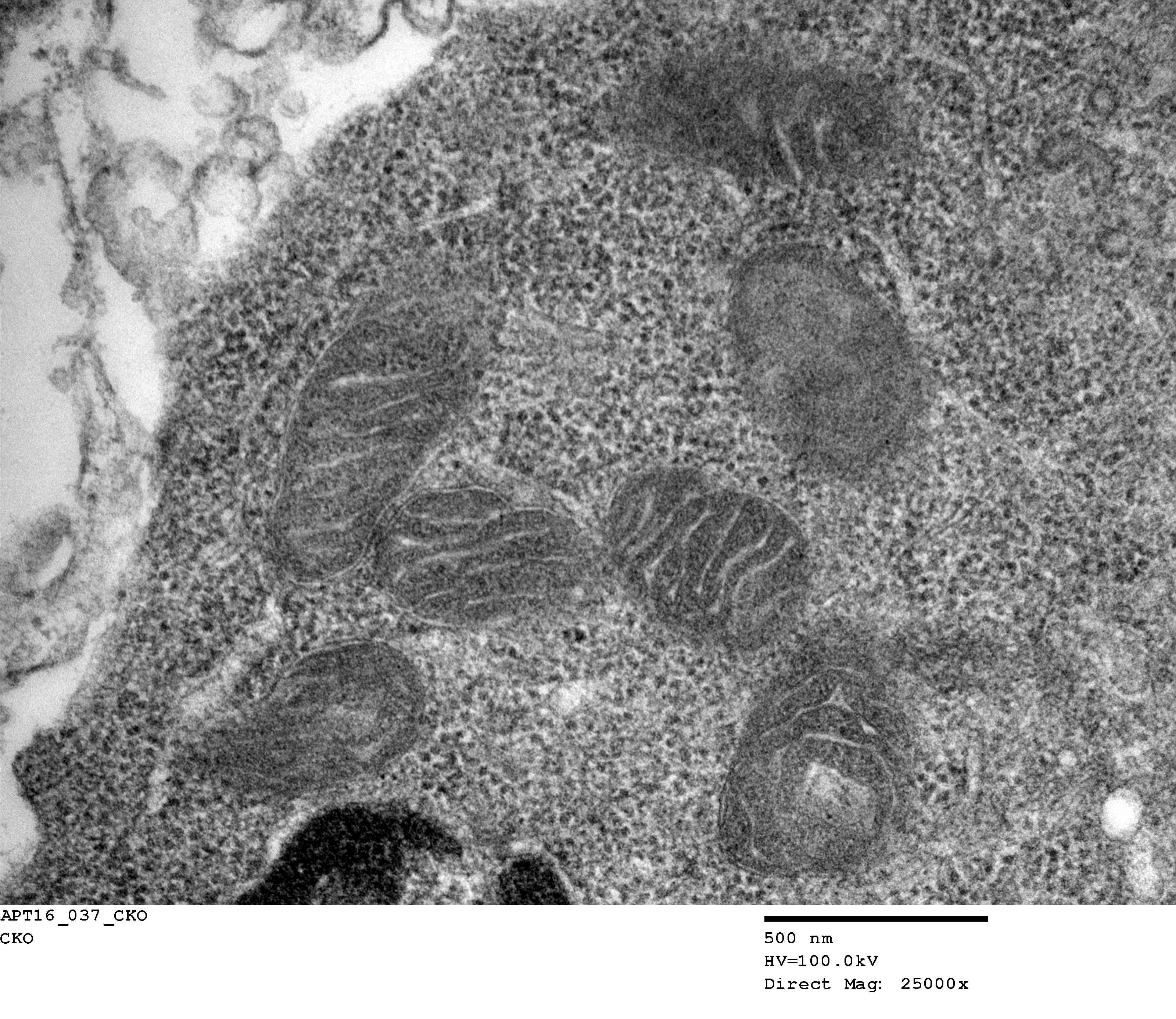

Supplement: Figure 3—source data 5. [file elife-66703-fig3-data5.zip › miR-146b CKO EM Pt 2 Fig 3ABDE/APT16_037_CKO.TIF]

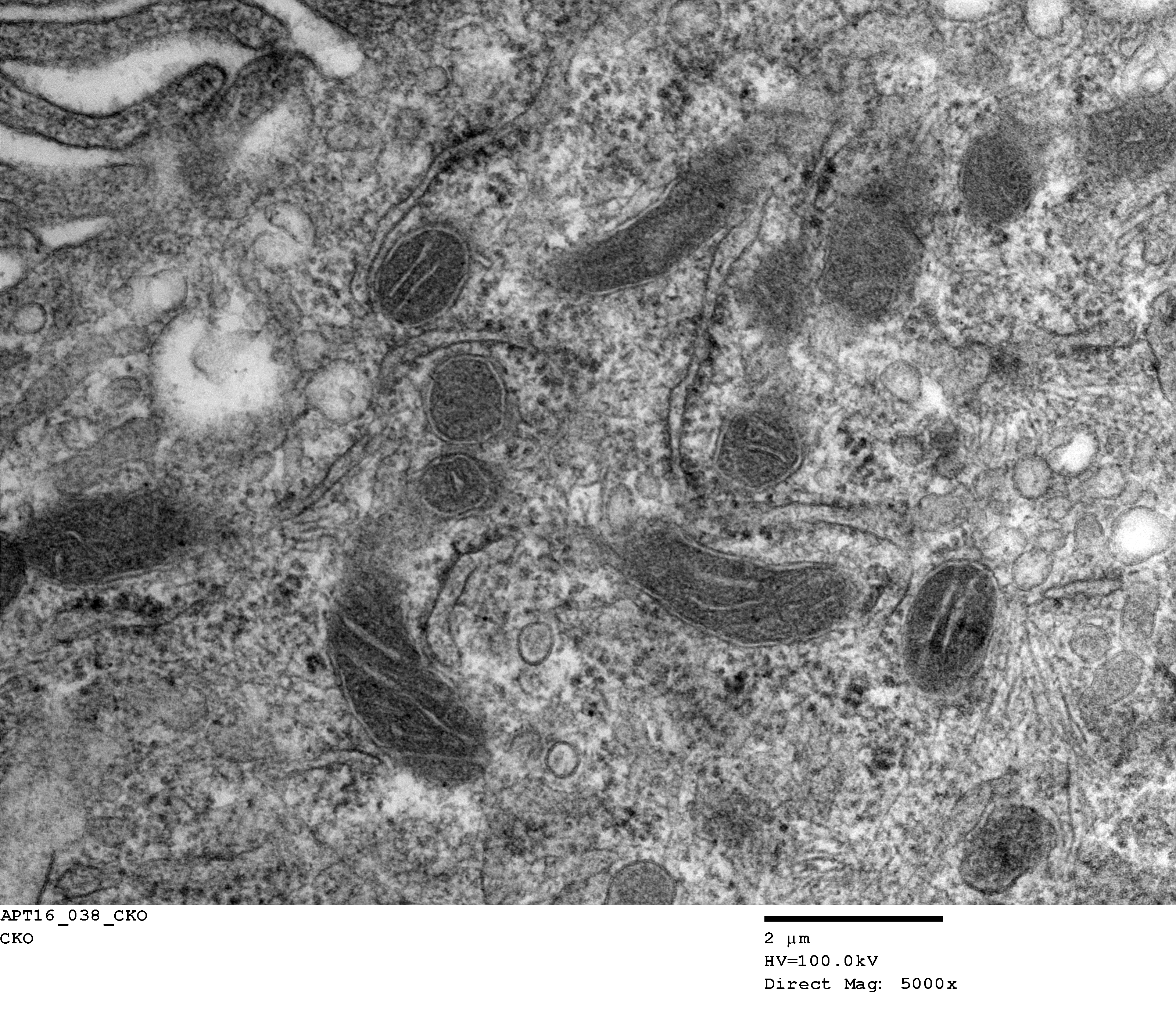

Supplement: Figure 3—source data 5. [file elife-66703-fig3-data5.zip › miR-146b CKO EM Pt 2 Fig 3ABDE/APT16_038_CKO.TIF]

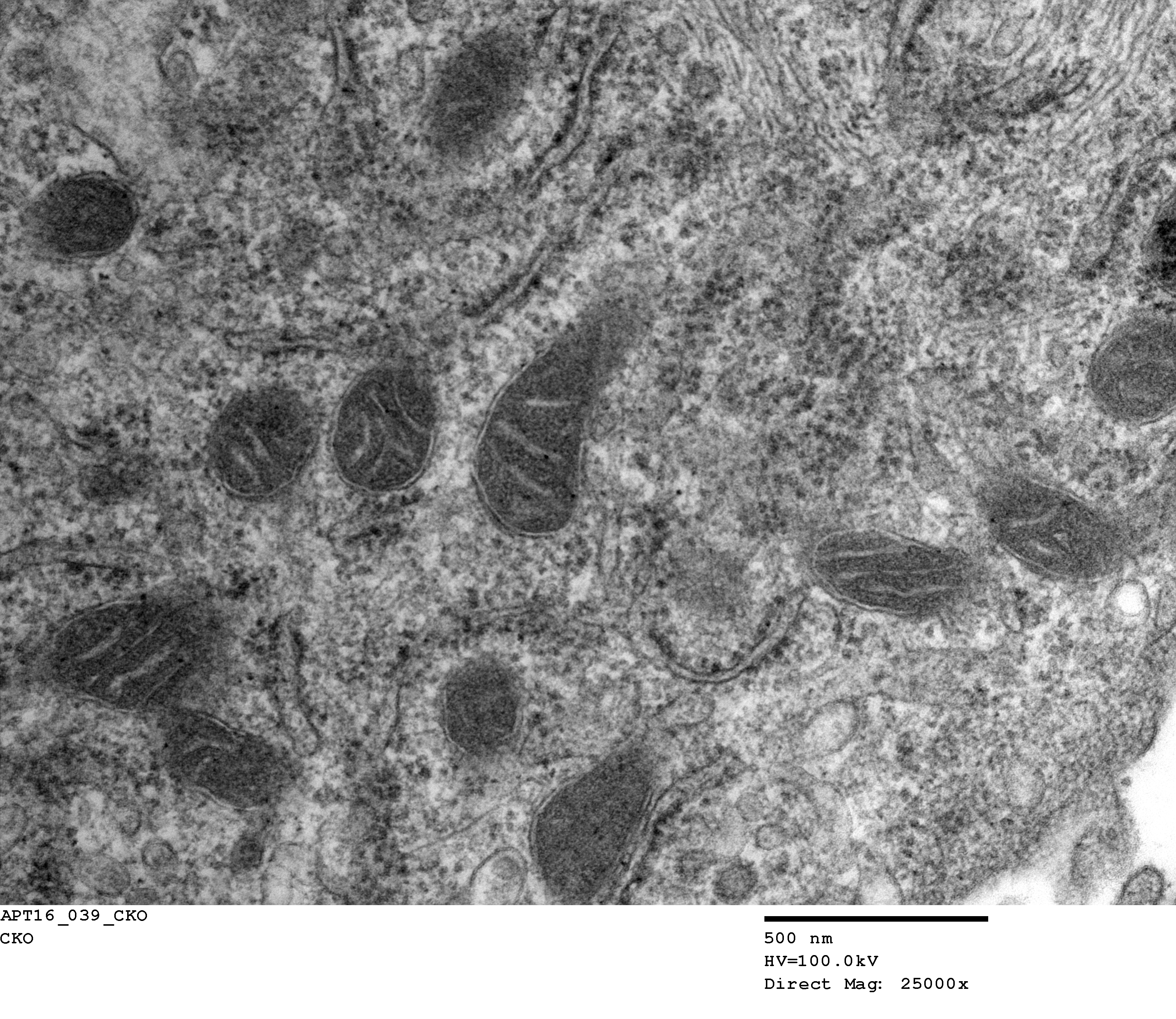

Supplement: Figure 3—source data 5. [file elife-66703-fig3-data5.zip › miR-146b CKO EM Pt 2 Fig 3ABDE/APT16_039_CKO.TIF]

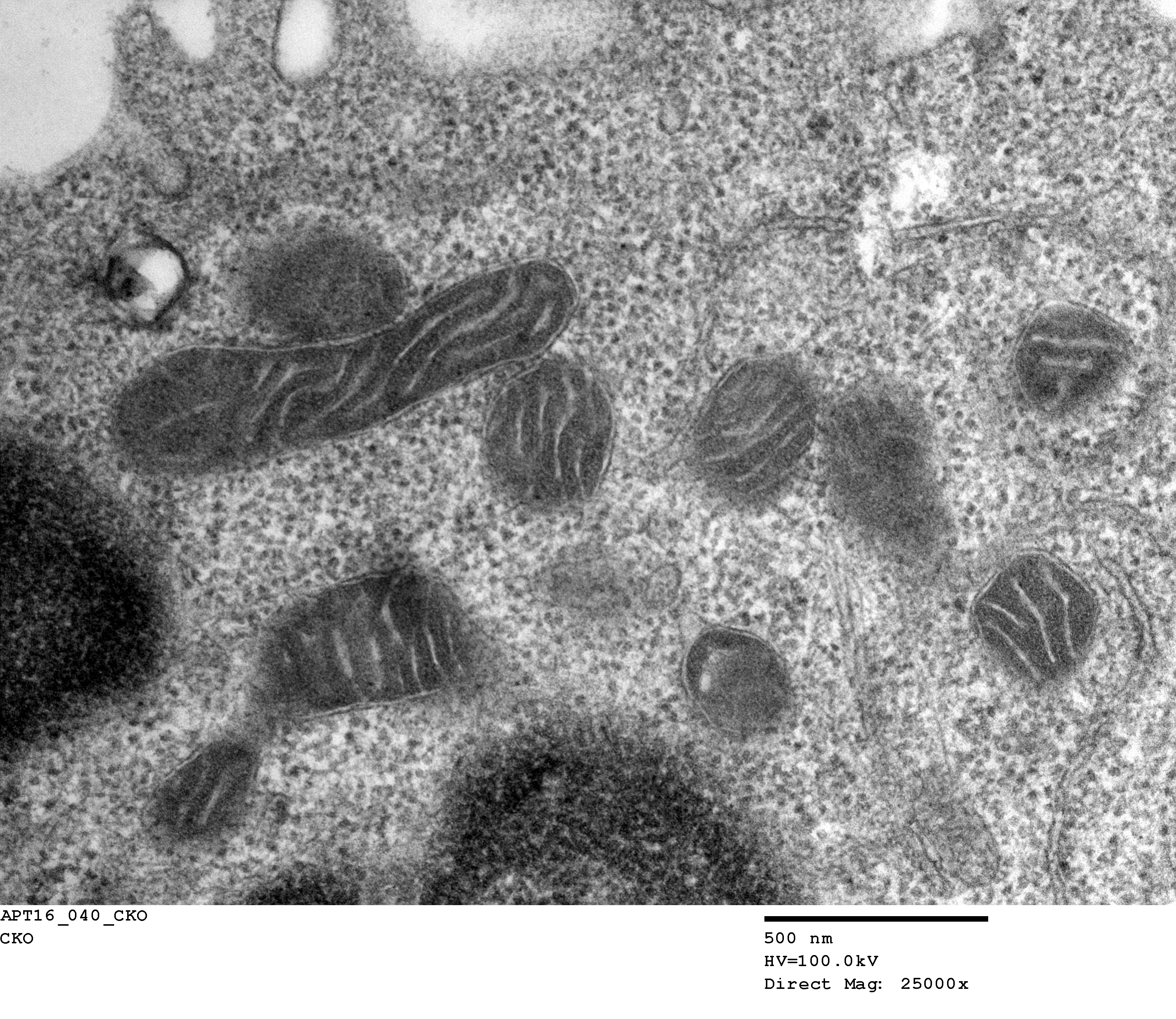

Supplement: Figure 3—source data 5. [file elife-66703-fig3-data5.zip › miR-146b CKO EM Pt 2 Fig 3ABDE/APT16_040_CKO.TIF]

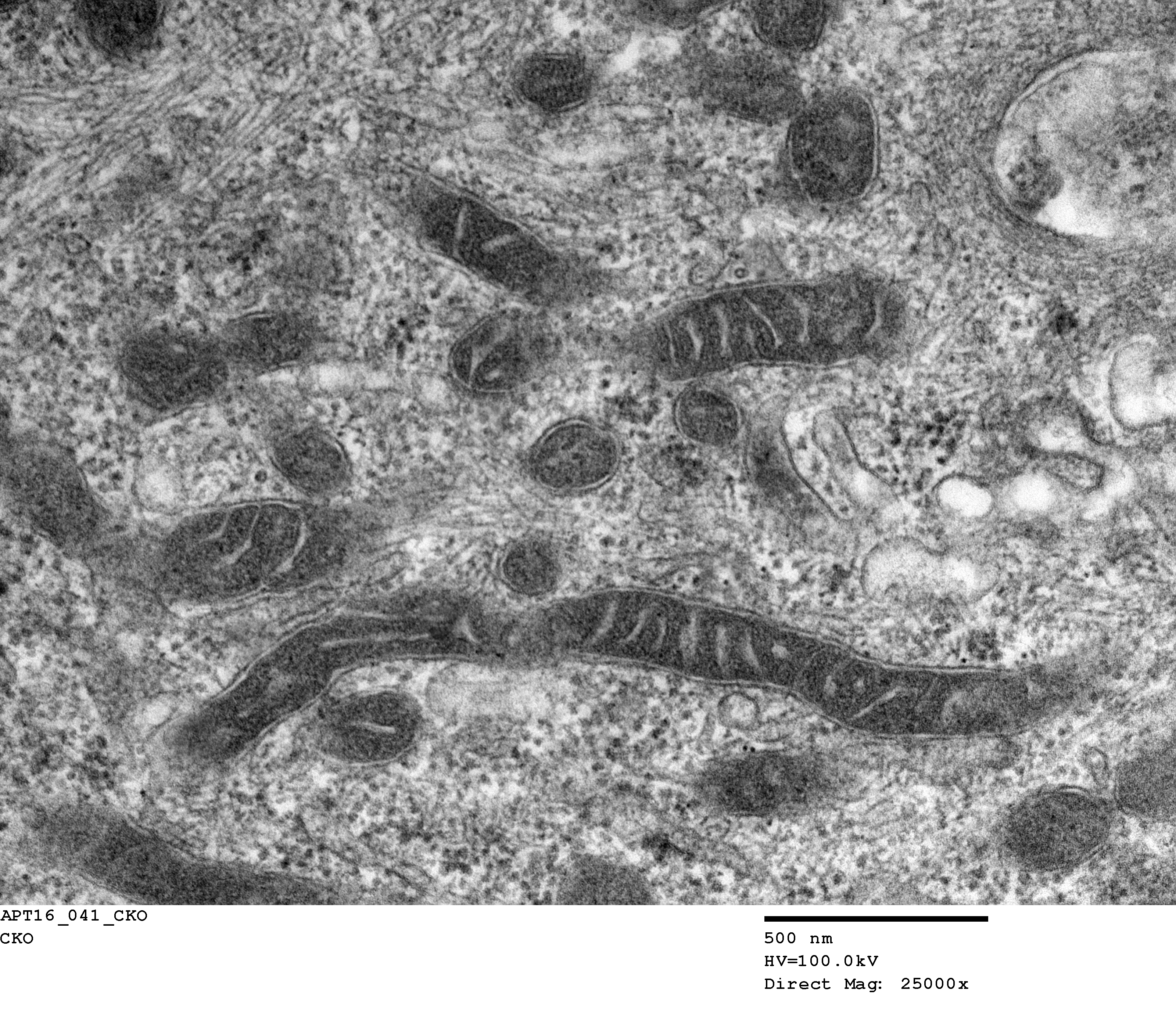

Supplement: Figure 3—source data 5. [file elife-66703-fig3-data5.zip › miR-146b CKO EM Pt 2 Fig 3ABDE/APT16_041_CKO.TIF]

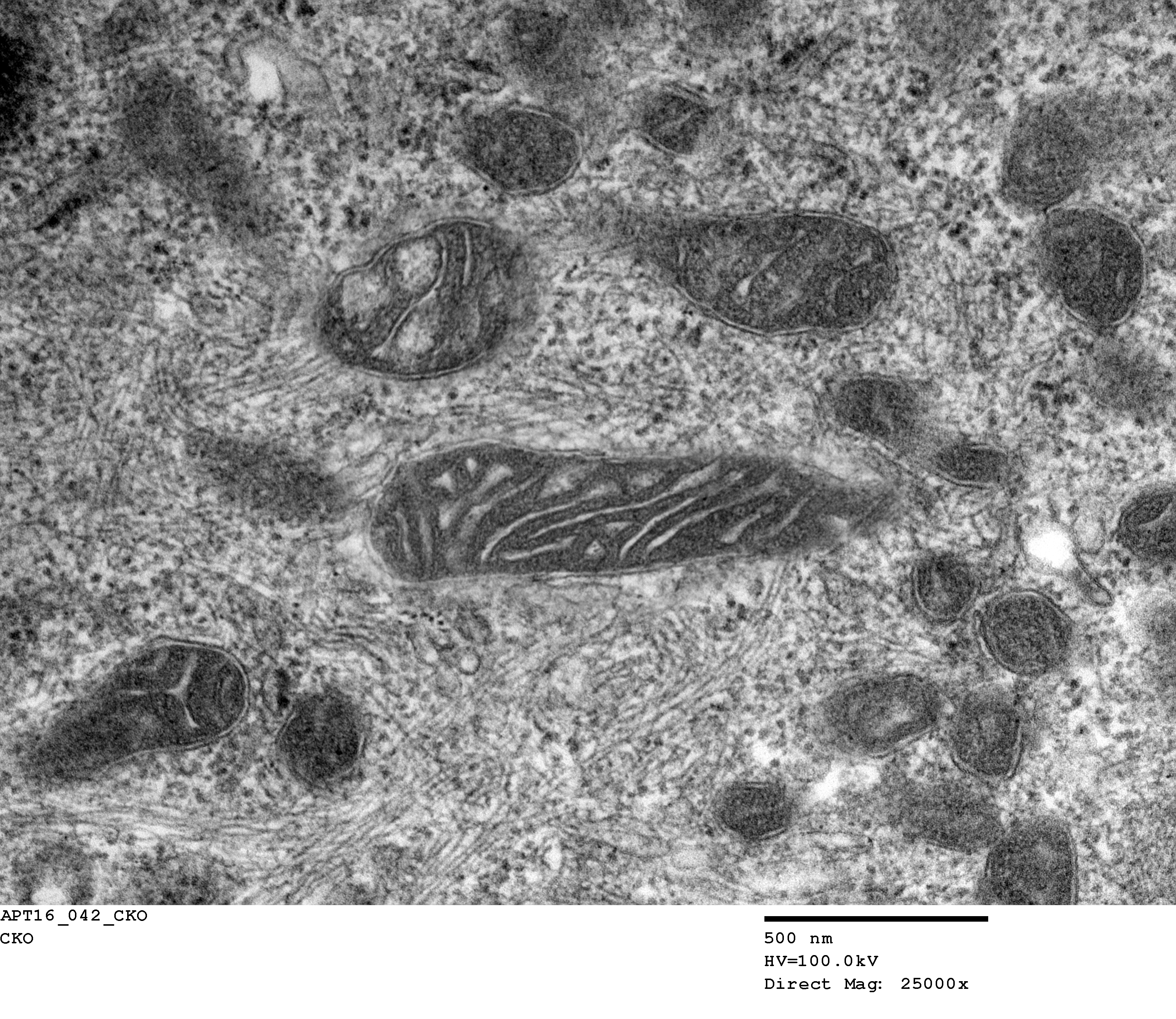

Supplement: Figure 3—source data 5. [file elife-66703-fig3-data5.zip › miR-146b CKO EM Pt 2 Fig 3ABDE/APT16_042_CKO.TIF]

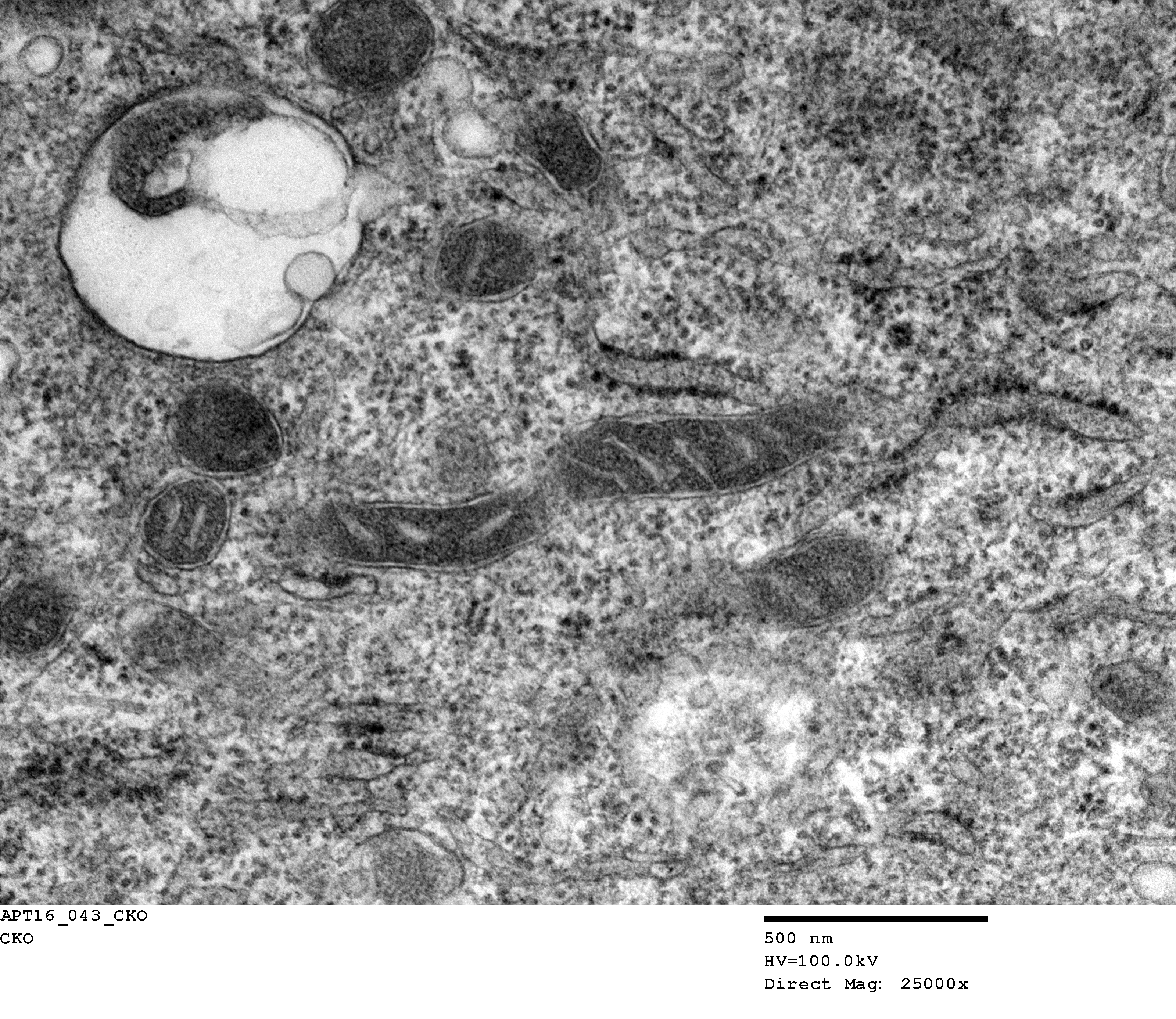

Supplement: Figure 3—source data 5. [file elife-66703-fig3-data5.zip › miR-146b CKO EM Pt 2 Fig 3ABDE/APT16_043_CKO.TIF]

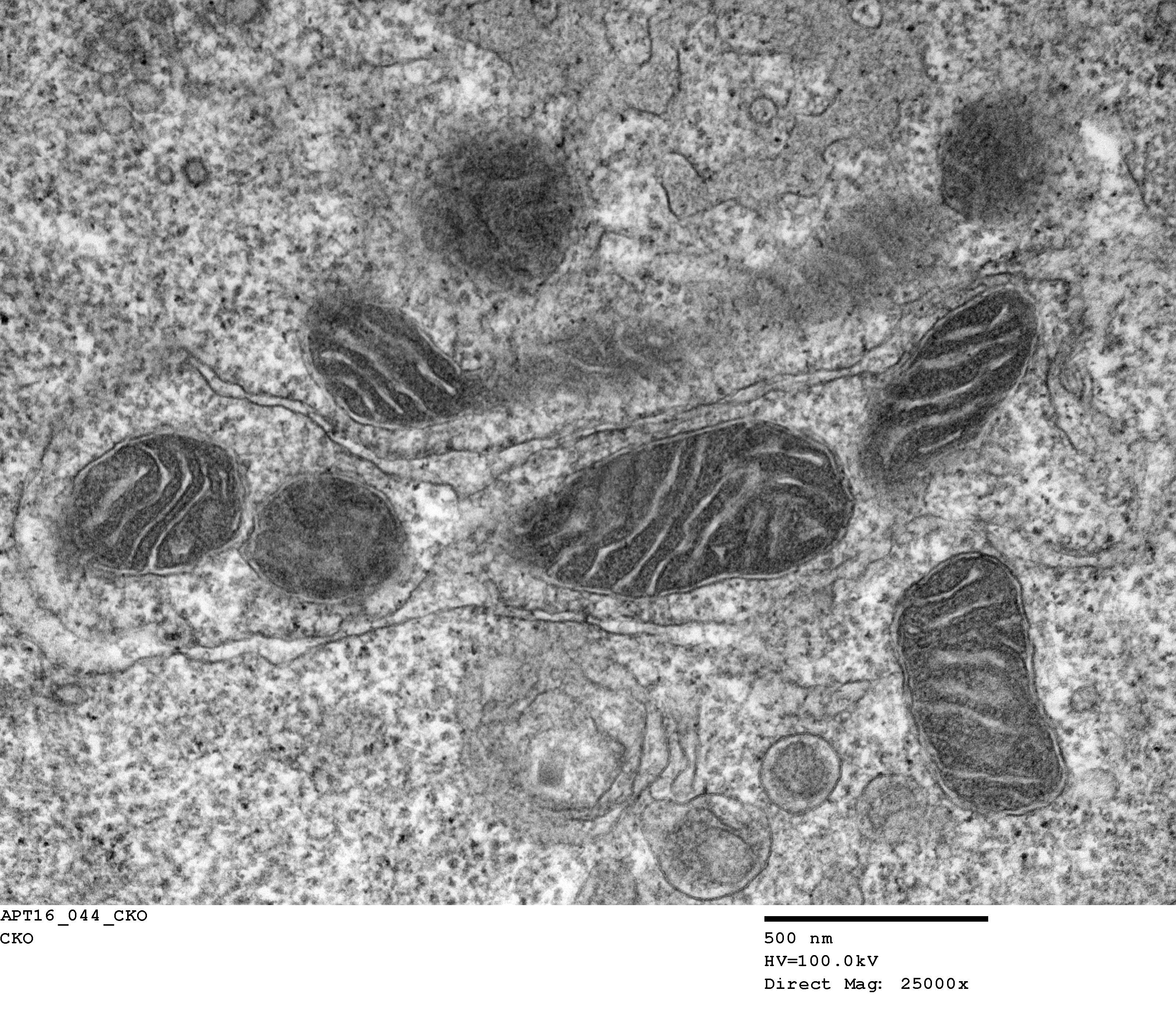

Supplement: Figure 3—source data 5. [file elife-66703-fig3-data5.zip › miR-146b CKO EM Pt 2 Fig 3ABDE/APT16_044_CKO.TIF]

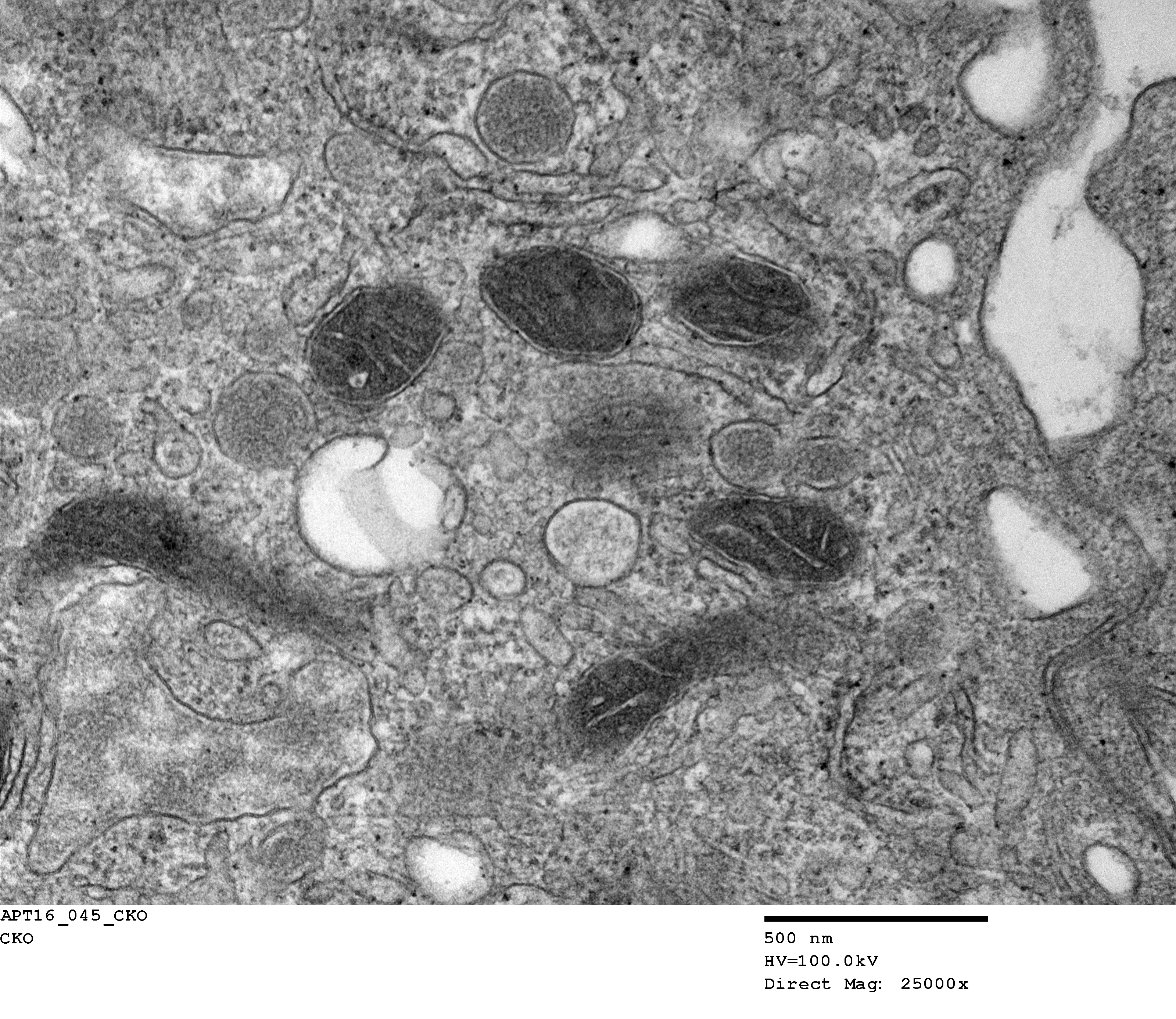

Supplement: Figure 3—source data 5. [file elife-66703-fig3-data5.zip › miR-146b CKO EM Pt 2 Fig 3ABDE/APT16_045_CKO.TIF]
