# Supplementary material for: Loss of Mir146b with aging contributes to inflammation and mitochondrial dysfunction in thioglycollate-elicited peritoneal macrophages
Source: eLife. 2021 Aug 23;10:e66703. doi: 10.7554/eLife.66703 (PMC8412946; doi:10.7554/eLife.66703)
Supplement: Supplementary file 1. [file elife-66703-supp1.docx]

| **Subcluster Pattern** | **Gene ID** |
| --- | --- |
| **a** | *AW112010*, *Ccl4, Ccl5, Clec4e, Cox6a2, Fabp3, Fcna, Gdf15, Ggh, H2-Q7, Hp, Ier3, Ifitm3, Igfbp4, Il18bp, Irf7, Irg1, Isg15, Ly6e, Mcemp1, Nupr1, Pf4, Psmb10, Saa3, Sh3bp5, Slpi, Wfdc17, Zbp1* |
| **b** | *Apoe, Chchd10, Dusp23, Fdps, Gas5, Gm11808, Gm8730, Isyna1, Lyz2, Napsa,*  *Ramp1, Sdf2l1, Slamf7, Tmem37, Tnfsf13os, Trf, Tspan17* |
| **c** | *BC005537, Cd300ld, Clec7a, Coro1c, Dusp1, Emc1, Gm10263, Hist1h1c, Lpl, Lpp, Mdfic, Mir6236, Mrc1, Srsf5, Tfrc, Yam1* |
| **d** | *AA467197, AI606181, Arap2, Arhgap39, Arrdc3, Atp5l, Ccl8, Ccl9, Cd24a, Cenpa, Cgnl1, Chil3, Clec7a,Cmbl, Cpeb1, Dach1, Dag1, Dhcr24, Dnmt3aos, Erdr1, Ermard, Fabp7, Fam46c, Gm10036, Gm10175, Gm7536, Gpr183, Gpx3, Hist1h2ap, Hmgb2, Hopx, Ifi203, Kcnj2, Kctd12b, Lipo1, Lsp1, Lyz1, Maoa, Mir22hg, Nacc2, Nfkbid, Pdcd4, Ptgir, Ptprs, Rasgrp3, Rpl27-ps3, Rps4l, S100a4, Sgsm2, Slc7a2, Snhg9, Stk24, Suv420h2, Tnpo2, Traf6, Tsix, Wdfy1, Ypel2* |
| **e** | *Apbb2, Bhlhe41, Ccr1, Dcbld2, Dio2, Dlg3, Epas1, Fstl1, Gpc1, Gpr137b-ps, Gstm2, Ltbp3, Ly75, Mamdc2, Matk, Mcoln3, Mfge8, Mgll, Mmp12, Npy, Pdpn, Ptrh1, Rassf8, Sel1l3, Ttyh2, Wfs1,* |
