# Supplementary material for: Loss of Mir146b with aging contributes to inflammation and mitochondrial dysfunction in thioglycollate-elicited peritoneal macrophages
Source: eLife. 2021 Aug 23;10:e66703. doi: 10.7554/eLife.66703 (PMC8412946; doi:10.7554/eLife.66703)
Supplement: Supplementary file 2. [file elife-66703-supp2.docx]

| Primer | Sequence | Product Size (mw) |
| --- | --- | --- |
| Universal Neo F | TGC TCC TCG CGA GAA AGT ATC CAT CAT GGC | 300 |
| Universal Neo R | CGC CAA GCT CTT CAG CAA TAT CAC GGG TAG |  |
| Mir146b Neo F | ATA TCT GGC CCA CCA GGA ACA CAT | WT-624  KI w/NEO-2525  KI W/O NEO-731 |
| Mir146b Neo R | AGC CTC TGT GTG TGC TTG TGA CAT |  |
| LoxP F | TAA CGG CAT TAG CCA CCA CCT TCA | WT-214  KI-309  KIXCRE=NO BAND |
| LoxP R | TGG GTT ATG TAG GGA TCC TGG GTT | W/146B NEO F: WT-1841  KI-3837  KI NO NEO-2043  KI NO COND ARM-795 |
| Flp/o FWD | ATA GCA GCT TTG CTC CTT CG | Transgene-240bp |
| Flp/o REV | TGG CTC ATC ACC TTC CTC TT |  |
| Flp/o Internal FWD | CTA GGC CAC AGA ATT GAA AGA TCT | Internal Positive cntl-324bp |
| Flp/o Internal Rev | GTA GGT GGA AAT TCT AGC ATC ATC C |  |
| LysM Cre F | TAA CGG CAT TAG CCA CCT TCA | Cre positive-~700  Cre negative-no band |
| LysM Cre R | TGG GTT ATG TAG GGA TCC TGG GTT |  |
| General Cre F | GCA TTA CCG GTC GAT GCA ACG AGT GA | Cre positive-~408  Cre negative-no band |
| General Cre R | GAG TGA ACG AAC CTG GTC GAA ATC AG |  |
